# Supplementary figures and images for: Comparing statistical learning methods for complex trait prediction from gene expression
Source: PLoS One. 2025 Feb 11;20(2):e0317516. doi: 10.1371/journal.pone.0317516 (PMC11813155; doi:10.1371/journal.pone.0317516)

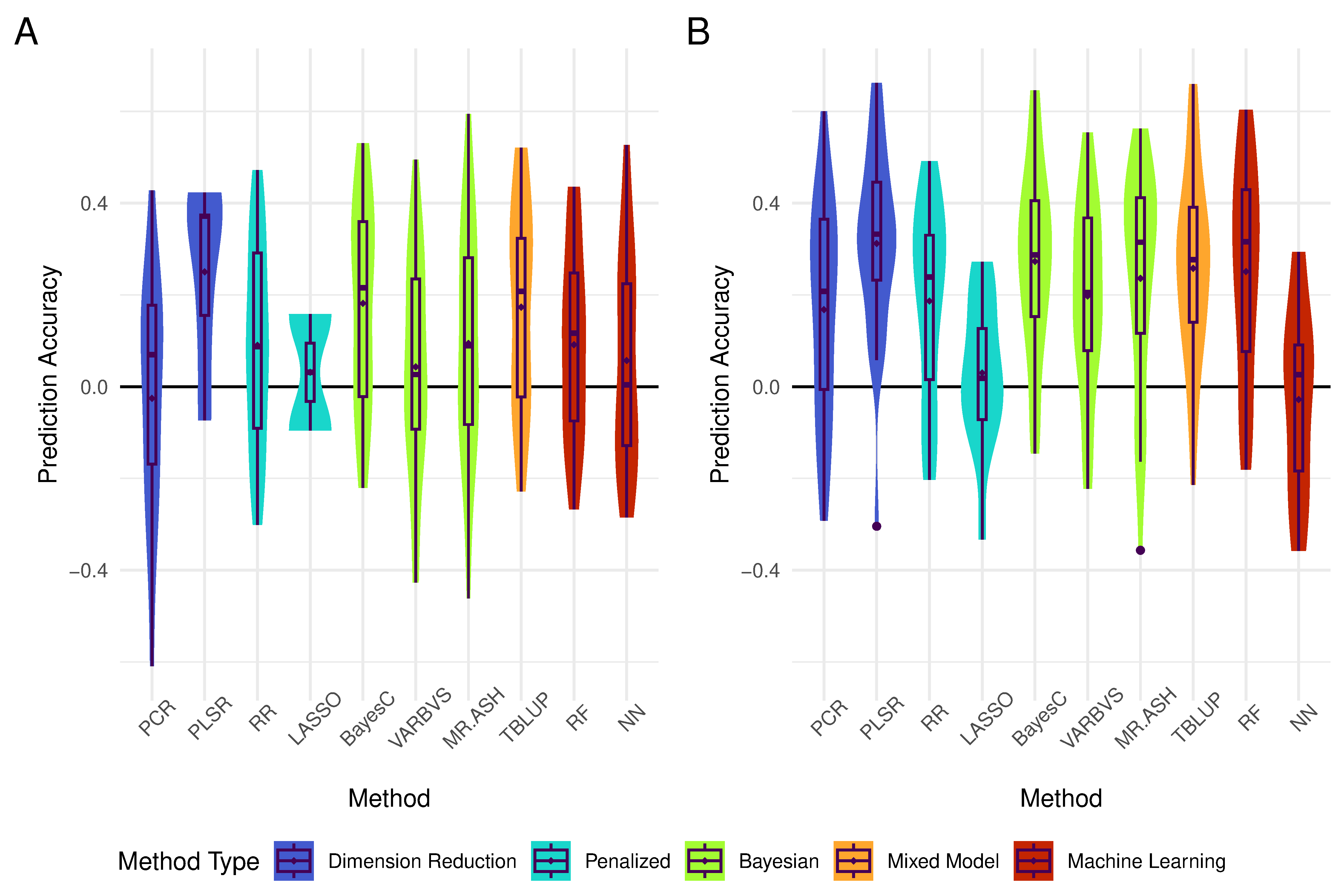

Supplement: S1 Fig — Prediction accuracy for 25 replicates in females (A) and males (B) for all standard methods. Methods are colored by family, where dimension reduction (blue), penalized regression (cyan), Bayesian regression (lime), linear mixed model (orange), and machine learning methods (red) are ordered from left to right. The mean correlation coefficient is denoted by diamonds. Outliers are denoted by circles. (TIF) [file pone.0317516.s001.tif]

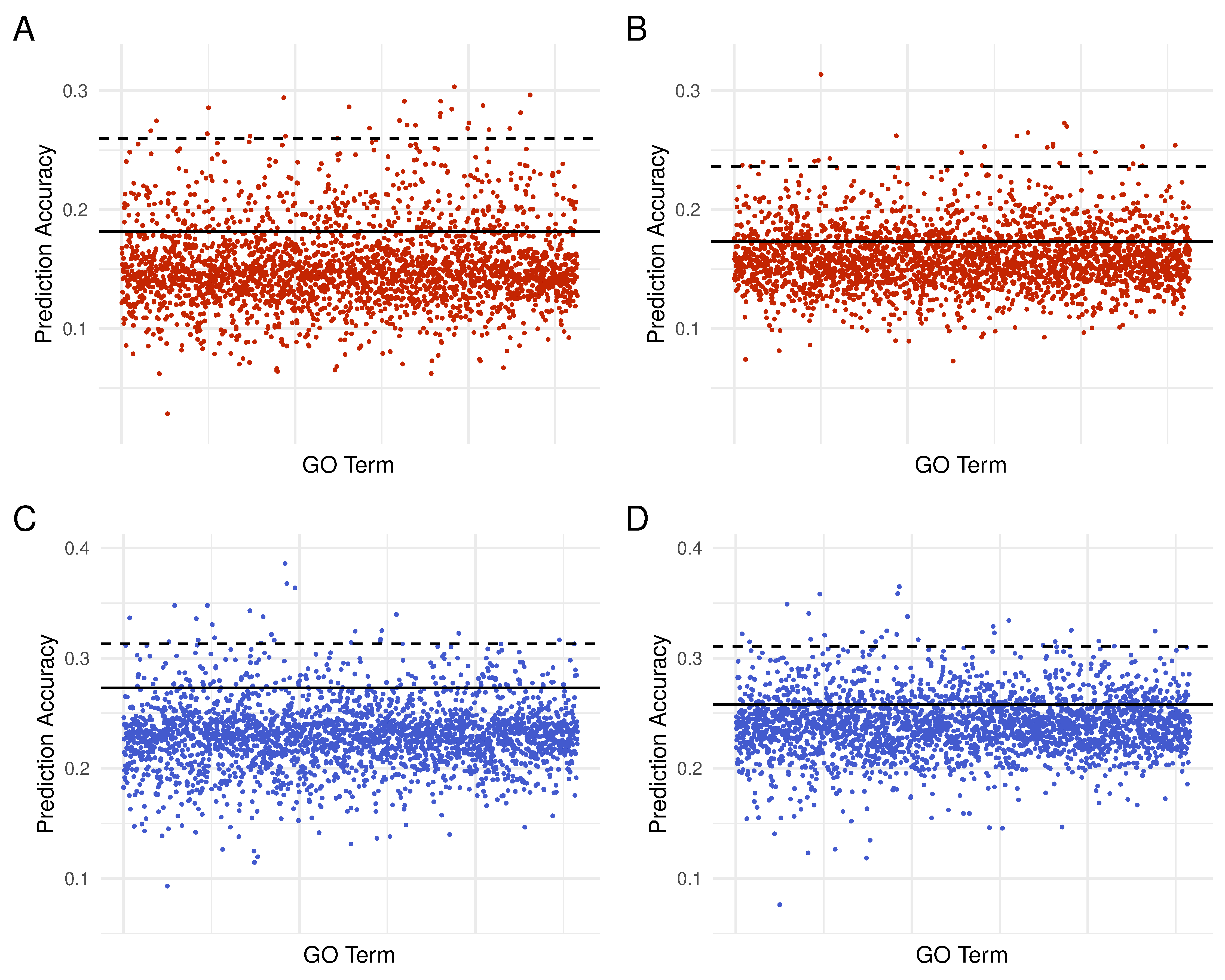

Supplement: S2 Fig — Prediction accuracy in the two sexes using GO-BayesC (A for females, C for males) and GO-TBLUP (B for females, D for males). Each dot represents the mean correlation between true and predicted phenotypes (r) across 25 replicates for a GO term. The solid line indicates the mean r from the respective standard method (i.e., BayesC and TBLUP). The dashed black line represents the 99th percentile of terms ranked by prediction accuracy. (TIF) [file pone.0317516.s002.tif]

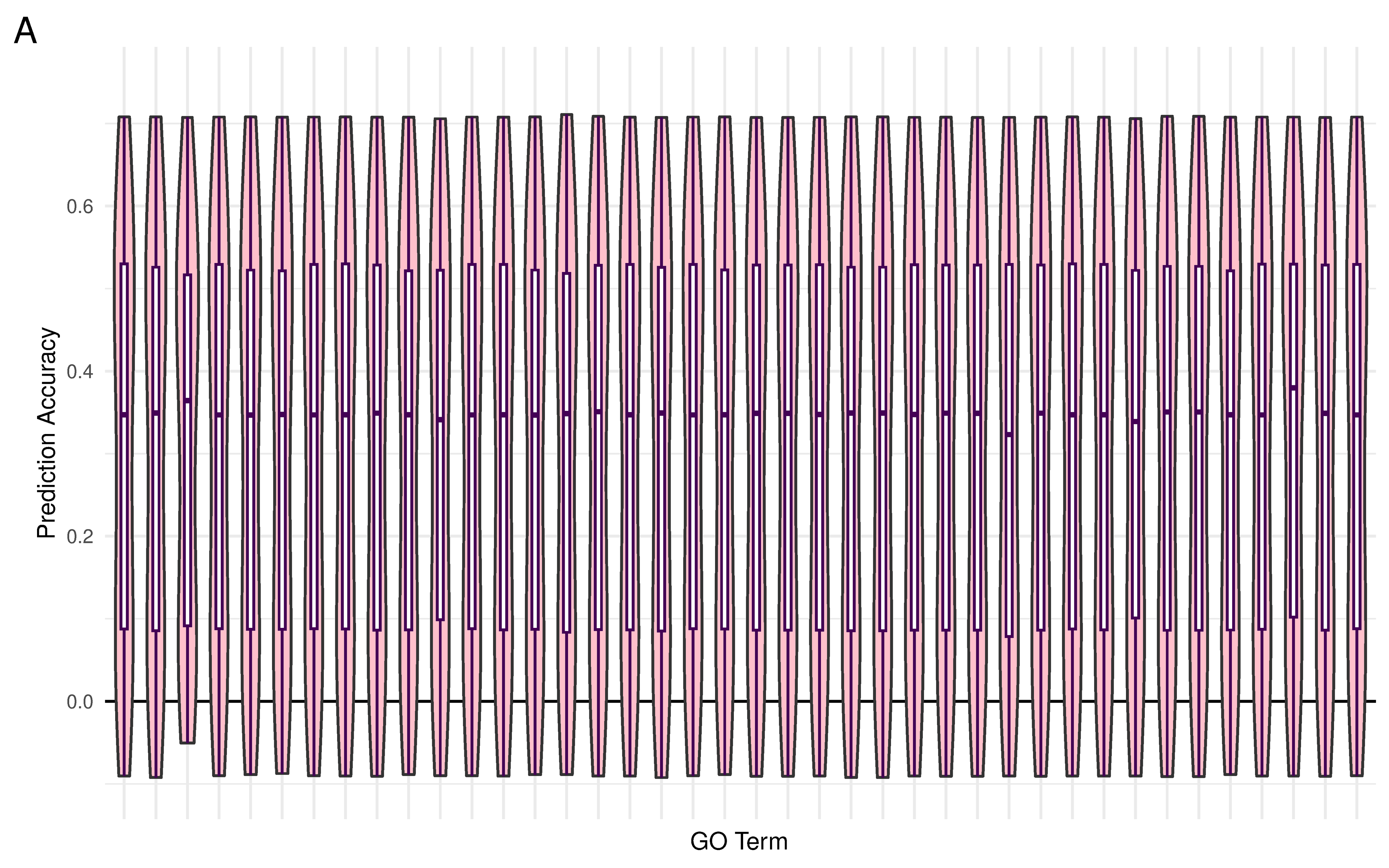

Supplement: S3 Fig — Violin plot comparison of Sparse Group Lasso results for top GO terms from GO-BayesC/GO-TBLUP along with randomly selected GO terms in females. (TIF) [file pone.0317516.s003.tif]

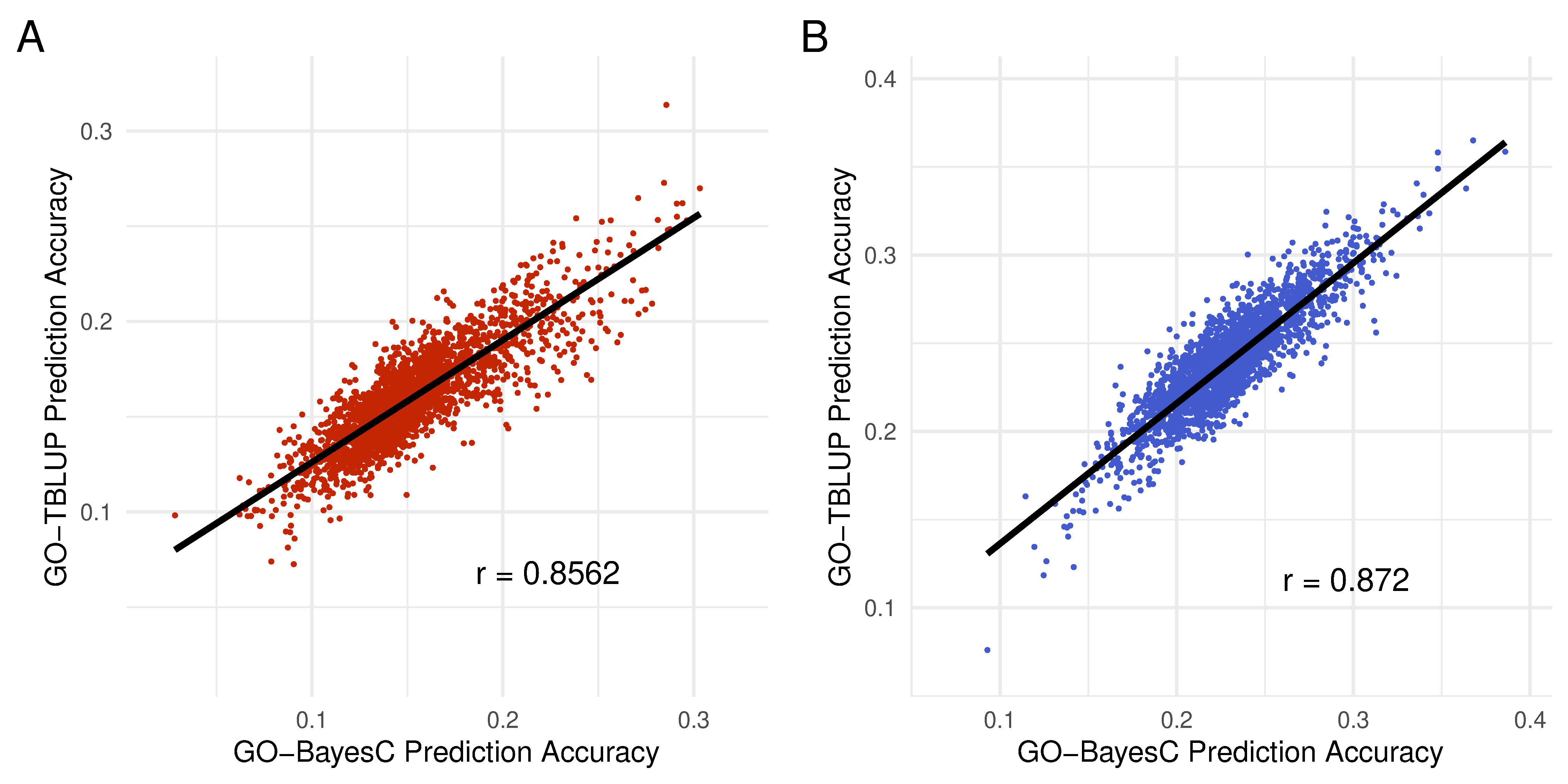

Supplement: S4 Fig — Prediction accuracy for all GO terms using GO-BayesC (x-axis) against GO-TBLUP (y-axis) for females (A) and males (B). The black line represents the line of least squares fit for each panel. (TIF) [file pone.0317516.s004.tif]

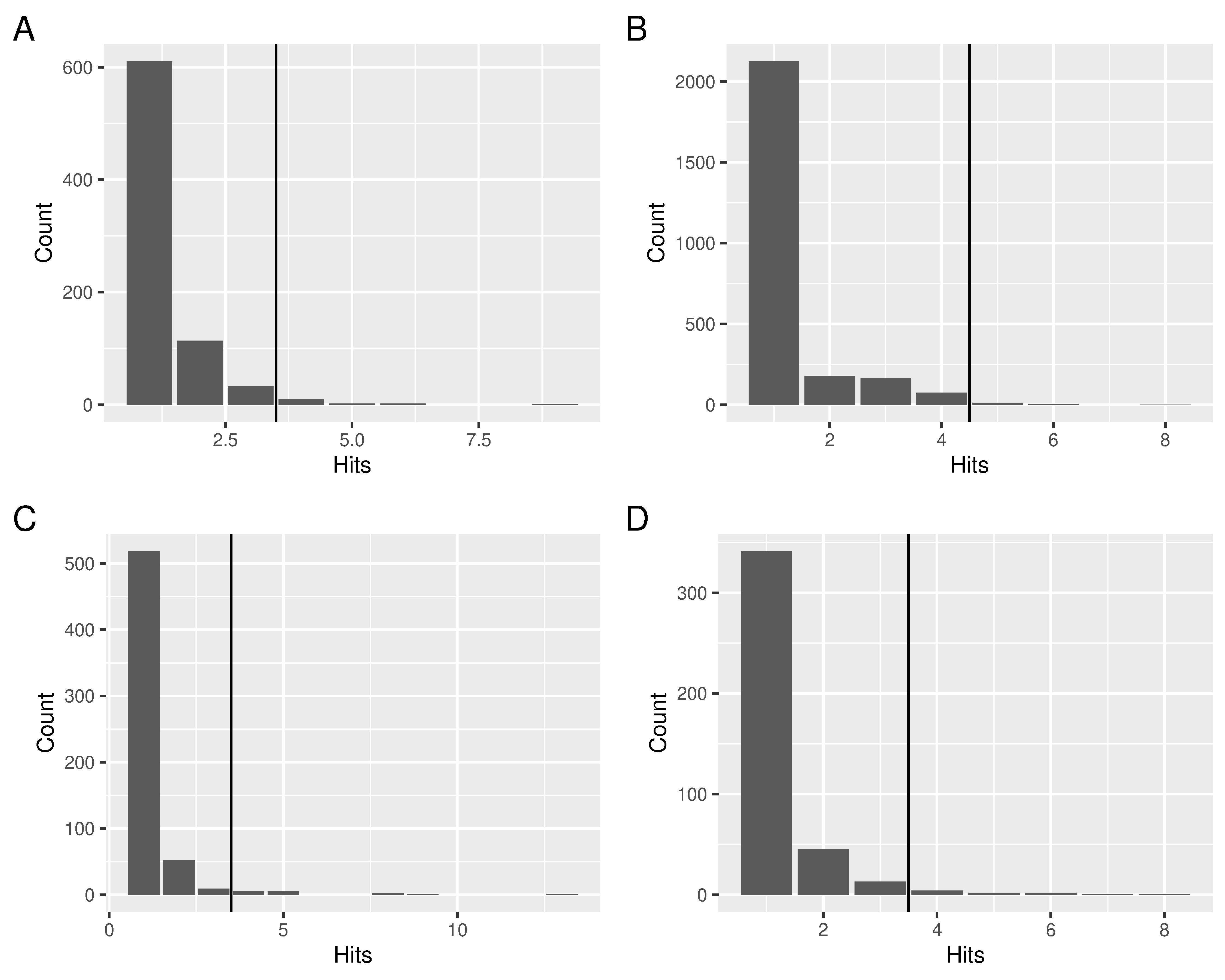

Supplement: S5 Fig — Distribution of number of overlapping genes for the top 1% of GO terms in the two sexes using GO-BayesC (A for females, C for males) and GO-TBLUP (B for females, D for males). The selection cutoff is marked by the vertical bar. (TIF) [file pone.0317516.s005.tif]

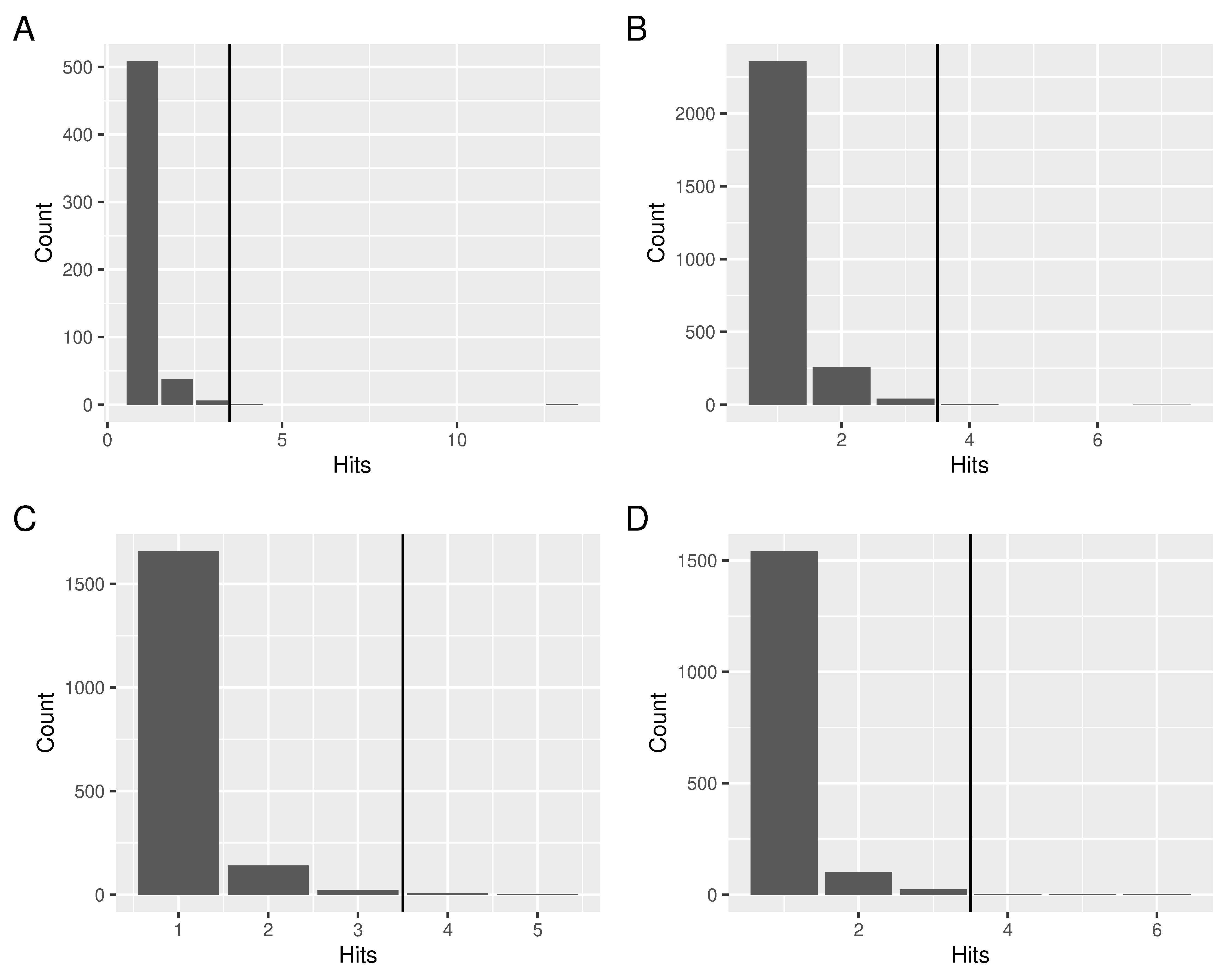

Supplement: S6 Fig — Distribution of number of overlapping genes for the top 1% of GO terms in the two sexes using GO-BayesC (A for females, C for males) and GO-TBLUP (B for females, D for males). The selection cutoff is marked by the vertical bar. (TIF) [file pone.0317516.s006.tif]
