# Supplementary material for: Comparing statistical learning methods for complex trait prediction from gene expression
Source: PLoS One. 2025 Feb 11;20(2):e0317516. doi: 10.1371/journal.pone.0317516 (PMC11813155; doi:10.1371/journal.pone.0317516)
Supplement: S1 Text — Detailed description of the genes identified in the gene analysis, including their relevance to the traits of interest. (PDF) [file pone.0317516.s015.pdf]

Comparing statistical learning methods for complex trait prediction from gene expression — S1 Text

Noah Klimkowski Arango[1,2], Fabio Morgante[1,2]

1 Center for Human Genetics, Clemson University, Greenwood, SC, USA
2 Department of Genetics and Biochemistry, Clemson University, Clemson, SC, USA

Gene Analysis Details

In females, GO-BayesC and GO-TBLUP found 5 genes in common out of the top 1% of GO terms for starvation resistance. These five genes are related to insulin signaling and lipid metabolism. Insulin signaling is involved in cell growth, feeding, carbohydrate metabolism, and many other traits critical for survival [1]. The insulin-like receptor (*InR*) is crucial for insulin signaling in carbohydrate metabolism. The adipokinetic hormone receptor (*AkhR*) is responsible for both carbohydrate and lipid metabolism signals. *AkhR* was shown to coexpress with *InR* on starvation-induced hyperactivity [2]. *Akt1* is the core kinase subunit of the insulin growth factor pathway and has been implicated in starvation resistance in a cancer study [3]. Aside from insulin signaling, the top two genes shared between methods have been implicated in starvation resistance. Epidermal growth factor receptor (*Egfr*) is important for normal cell growth, while overexpression of the receptor is a common route for cancer development [4]. Sevelda *et al.* showed that *Egfr* increased starvation resistance through interactions with *Akt1* [5]. *Erk7* is an extracellular kinase involved in the secretory system. *Erk7* downregulates secretion by triggering the destruction of endoplasmic reticulum exit sites under starvation conditions [6]. For startle response, only crumbs (*crb*), a gene involved in Notch regulation and photoreceptor morphogenesis, was shared between GO-BayesC and GO-TBLUP.

For starvation resistance, most of the genes found by GO-BayesC only are from the nucleoporin family (*mbo, Nup53, Nup54, Nup93-1, Nup93-2, Nup98-96, Nup153, Nup205*). Nucleoporin degradation can occur under starvation conditions to prevent the export of macromolecules [7]. The remaining genes are tangential to the *InR* signaling pathway. The adipokinetic hormone (*Akh*) makes a complex with its receptor *AkhR* previously described as part of the *InR* signaling pathway. Downstream in the pathway, a signal propagating kinase *Pi3K92E* was also found by GO-BayesC. For startle response, *aPKC* was the only other gene found by GO-BayesC. *aPKC* complexes with *crb* to promote neuroblast proliferation and renewal. For starvation resistance, eleven distinct genes found by GO-TBLUP only are involved in various complexes and pathways. Three of these genes are related to the Hippo, or Salvador-Warts-Hippo, signaling pathway [8] — the core signaling kinase hippo (*hpo*), a kinase further downstream (*ack*), and *Sik3*. *Sik3* is involved in balancing NADPH/NADP+, a set of key components for oxidative stress mitigation and cellular signaling [9]. *Sik3* is also part of the *InR* signaling pathway that negatively regulates Hippo signaling. Another top gene (*pdk1*) is part of the *InR* signaling pathway and is responsible for inhibiting apoptosis during embryonic development. In flies, activin signaling responds to carbohydrate levels in the gut by increasing carbohydrase expression [10]. Additionally, activin signaling increases starvation resistance for neuronal cells [11]. The products of two genes, *put* and *babo*, form a complex with an activin-like ligand to initiate the activin signaling pathway [12]. Hemipterous (*hep*) is a kinase involved in imaginal disc formation and cell proliferation. A study found that *hep* loss-of-function mutant flies have reduced energy stores and lower starvation resistance. [13] *hep* has also been

implicated in obesity and insulin resistance by activating the JNK signaling pathway [14]. For startle response, three genes were found by GO-TBLUP only. These genes are involved in olfactory receptors (*Orco*), circadian signalling (*dco*), and locomotion (*park*), and have implications in startle responses [15–17].

In males, GO-BayesC and GO-TBLUP found eight genes in common out of the top 1% of GO terms for starvation resistance. The two major categories that emerge from these genes are carbohydrate metabolism and cell polarization. For carbohydrate metabolism, the insulin-like receptor (*InR*) and an insulin-like peptide (*Ilp2*) are key components of *InR* signaling. *InR* is the only gene that was found by both GO methods in both sexes. The remaining genes are all related to cell polarization. The Crumbs complex has three genes (*crb, sdt, Patj*), while the PAR complex has two genes (*aPKC* and *par-6*) from both methods. Both complexes are highly conserved regulators of apico-basal cell formation [18]. The PAR complex negatively regulates the *InR* signaling pathway [19]. Outside of these complexes, *PDZ-GEF* is a guanine exchange factor involved in epithelial cell polarization through a separate mechanism [20]. The startle response analysis yielded two prevalent genes in common between GO-BayesC and GO-TBLUP — *Idgf2* is involved in stress response while *stg* is involved in cell cycle progression.

Six genes were found by GO-BayesC only in the top 1% of GO terms from males for starvation resistance. Three genes are related to the PAR complex. Bazooka (*baz*) is a core component of the PAR complex while two genes, *scrib* and *dlg1*, form a conserved complex with *lgl* that regulates cell migration with the PAR complex [21]. The product of *Moe* competes with PAR complex component *aPKC* to regulate the Crumbs complex [22]. *AkhR* was found uniquely by GO-BayesC. As previously described, this gene is involved in carbohydrate and lipid metabolism signaling processes [2]. Another gene, shotgun(*shg*), is downstream in the *Egfr* signaling pathway [23]. As previously described, *Egfr* promotes starvation resistance through *Akt1* interactions [5]. For startle response, GO-BayesC found five *Idgf* genes, in addition to *Idgf2*, and three neuromuscular genes (*wg*, *Ptp99A*, and *Ptp69D*). Only two genes were found uniquely by GO-TBLUP in males for starvation resistance. One is a desaturase (*Desat1*) that synthesizes fatty acid molecules. *Desat1* has been shown to induce cell autophagy under starvation conditions [24]. The other, *Ras85D*, is an oncogenic cell growth promoter. Downregulating *Ras85D* has been shown to improve starvation resistance by limiting growth signals [25]. For startle response, GO-TBLUP uniquely found *Notch*, *Cul3*, and *Dl*. *Notch* and *Dl* are involved in nervous system development via Notch signalling and have been associated with disruptions in startle response [26]. *Cul3* is also involved in neuron growth for sensory organ development.

References

1. Strilbytska OM, Semaniuk UV, Storey KB, Yurkevych IS, Lushchak O. Insulin signaling in intestinal stem and progenitor cells as an important determinant of physiological and metabolic traits in Drosophila. Cells. 2020;9(4):803.

2. Yu Y, Huang R, Ye J, Zhang V, Wu C, Cheng G, et al. Regulation of starvation-induced hyperactivity by insulin and glucagon signaling in adult Drosophila. Elife. 2016;5:e15693.

3. Clark AS, West K, Streicher S, Dennis PA. Constitutive and inducible Akt activity promotes resistance to chemotherapy, trastuzumab, or tamoxifen in breast cancer cells. Molecular cancer therapeutics. 2002;1(9):707–717.

4. Tan X, Lambert PF, Rapraeger AC, Anderson RA. Stress-induced EGFR trafficking: mechanisms, functions, and therapeutic implications. Trends in cell biology. 2016;26(5):352–366.

5. Sevelda F, Mayr L, Kubista B, Lötsch D, van Schoonhoven S, Windhager R, et al. EGFR is not a major driver for osteosarcoma cell growth in vitro but contributes to starvation and chemotherapy resistance. Journal of Experimental & Clinical Cancer Research. 2015;34:1–12.

6. Zacharogianni M, Kondylis V, Tang Y, Farhan H, Xanthakis D, Fuchs F, et al. ERK7 is a negative regulator of protein secretion in response to amino-acid starvation by modulating Sec16 membrane association. The EMBO journal. 2011;30(18):3684–3700.

7. Lee CW, Wilfling F, Ronchi P, Allegretti M, Mosalaganti S, Jentsch S, et al. Selective autophagy degrades nuclear pore complexes. Nature cell biology. 2020;22(2):159–166.

8. Harvey K, Tapon N. The Salvador–Warts–Hippo pathway—an emerging tumour-suppressor network. Nature Reviews Cancer. 2007;7(3):182–191.

9. Agledal L, Niere M, Ziegler M. The phosphate makes a difference: cellular functions of NADP. Redox Report. 2010;15(1):2–10.

10. Sleiman MSB, Schüpfer F, Lemaitre B, et al. Transforming growth factor β/activin signaling functions as a sugar-sensing feedback loop to regulate digestive enzyme expression. Cell reports. 2014;9(1):336–348.

11. Chng WbA, Koch R, Li X, Kondo S, Nagoshi E, Lemaitre B. Transforming Growth Factor β/Activin signaling in neurons increases susceptibility to starvation. PloS one. 2017;12(10):e0187054.

12. Song W, Cheng D, Hong S, Sappe B, Hu Y, Wei N, et al. Midgut-derived activin regulates glucagon-like action in the fat body and glycemic control. Cell metabolism. 2017;25(2):386–399.

13. Hull-Thompson J, Muffat J, Sanchez D, Walker DW, Benzer S, Ganfornina MD, et al. Control of metabolic homeostasis by stress signaling is mediated by the lipocalin NLaz. PLoS genetics. 2009;5(4):e1000460.

14. Solinas G, Becattini B. JNK at the crossroad of obesity, insulin resistance, and cell stress response. Molecular metabolism. 2017;6(2):174–184.

15. Eddison M, Belay AT, Sokolowski MB, Heberlein U. A genetic screen for olfactory habituation mutations in Drosophila: analysis of novel foraging alleles and an underlying neural circuit. PLoS One. 2012;7(12):e51684.

16. Chabot CC, Taylor DH. Circadian modulation of the rat acoustic startle response. Behavioral neuroscience. 1992;106(5):846.

17. Von Coelln R, Thomas B, Savitt JM, Lim KL, Sasaki M, Hess EJ, et al. Loss of locus coeruleus neurons and reduced startle in parkin null mice. Proceedings of the National Academy of Sciences. 2004;101(29):10744–10749.

18. Brown EB, Slocumb ME, Szuperak M, Kerbs A, Gibbs AG, Kayser MS, et al. Starvation resistance is associated with developmentally specified changes in sleep, feeding and metabolic rate. Journal of Experimental Biology. 2019;222(3):jeb191049.

19. Weyrich P, Kapp K, Niederfellner G, Melzer M, Lehmann R, Häring HU, et al. Partitioning-defective protein 6 regulates insulin-dependent glycogen synthesis via atypical protein kinase C. Molecular Endocrinology. 2004;18(5):1287–1300.

20. Consonni SV, Brouwer PM, van Slobbe ES, Bos JL. The PDZ domain of the guanine nucleotide exchange factor PDZGEF directs binding to phosphatidic acid during brush border formation. PLoS One. 2014;9(5):e98253.

21. Humbert PO, Dow LE, Russell SM. The Scribble and Par complexes in polarity and migration: friends or foes? Trends in cell biology. 2006;16(12):622–630.

22. Sherrard KM, Fehon RG. The transmembrane protein Crumbs displays complex dynamics during follicular morphogenesis and is regulated competitively by Moesin and aPKC. Development. 2015;142(10):1869–1878.

23. O'Keefe DD, Prober DA, Moyle PS, Rickoll WL, Edgar BA. Egfr/Ras signaling regulates DE-cadherin/Shotgun localization to control vein morphogenesis in the Drosophila wing. Developmental biology. 2007;311(1):25–39.

24. Paiardi C, Mirzoyan Z, Zola S, Parisi F, Vingiani A, Pasini ME, et al. The stearoyl-CoA desaturase-1 (Desat1) in Drosophila cooperated with Myc to induce autophagy and growth, a potential new link to tumor survival. Genes. 2017;8(5):131.

25. Kučerová L, Kubrak OI, Bengtsson JM, Strnad H, Nylin S, Theopold U, et al. Slowed aging during reproductive dormancy is reflected in genome-wide transcriptome changes in Drosophila melanogaster. BMC genomics. 2016;17:1–25.

26. Sargin D, Botly LC, Higgs G, Marsolais A, Frankland PW, Egan SE, et al. Reprint of: Disrupting Jagged1–Notch signaling impairs spatial memory formation in adult mice. Neurobiology of learning and memory. 2013;105:20–30.
