# Supplementary material for: Comparing statistical learning methods for complex trait prediction from gene expression
Source: PLoS One. 2025 Feb 11;20(2):e0317516. doi: 10.1371/journal.pone.0317516 (PMC11813155; doi:10.1371/journal.pone.0317516)
Supplement: Attachment — Submitted filename: PONE-D-24-23903 review.pdf [file pone.0317516.s016.pdf]

PLOS ONE

Comparing statistical learning methods for complex trait prediction from gene expression

--Manuscript Draft--

**Manuscript Number:**	PONE-D-24-23903
**Article Type:**	Research Article
**Full Title:**	Comparing statistical learning methods for complex trait prediction from gene expression
**Short Title:**	Transcriptomic prediction of complex traits
**Corresponding Author:**	Fabio Morgante<br>Clemson University<br>Greenwood, UNITED STATES
**Keywords:**	Transcriptomic prediction; prediction of complex traits; Bayesian regression; penalized regression; machine learning; gene ontology
**Abstract:**	Accurate prediction of complex traits is an important task in quantitative genetics that has become increasingly relevant for personalized medicine. Genotypes have traditionally been used for trait prediction using a variety of methods such as mixed models, Bayesian methods, penalized regressions, dimension reductions, and machine learning methods. Recent studies have shown that gene expression levels can produce higher prediction accuracy than genotypes. However, only a few prediction methods were used in these studies. Thus, a comprehensive assessment of methods is needed to fully evaluate the potential of gene expression as a predictor of complex trait phenotypes. Here, we used data from the Drosophila Genetic Reference Panel (DGRP) to compare the ability of several existing statistical learning methods to predict starvation resistance from gene expression in the two sexes separately. The methods considered differ in assumptions about the distribution of gene effect sizes – ranging from models that assume that every gene affects the trait to more sparse models – and their ability to capture gene-gene interactions. We also used functional annotation (i.e., Gene Ontology (GO)) as an external source of biological information to inform prediction models. The results show that differences in prediction accuracy between methods exist, although they are generally not large. Methods performing variable selection gave higher accuracy in females while methods assuming a more polygenic architecture performed better in males. Incorporating GO annotations further improved prediction accuracy for a few GO terms of biological significance. Biological significance extended to the genes underlying highly predictive GO terms with different genes emerging between sexes. Notably, the Insulin-like Receptor (InR) was prevalent across methods and sexes. Our results confirmed the potential of transcriptomic prediction and highlighted the importance of selecting appropriate methods and strategies in order to achieve accurate predictions.
**Order of Authors:**	Noah Klimkowski Arango
	Fabio Morgante
**Additional Information:**	
**Question**	**Response**
**Financial Disclosure**<br><br>Enter a financial disclosure statement that describes the sources of funding for the work included in this submission. Review the submission guidelines for detailed requirements. View published research articles from *PLOS ONE* for specific examples.	Yes

Powered by Editorial Manager® and ProduXion Manager® from Aries Systems Corporation

This statement is required for submission and **will appear in the published article** if the submission is accepted. Please make sure it is accurate.<br><br>**Funded studies**<br>Enter a statement with the following details:<br>• Initials of the authors who received each award<br>• Grant numbers awarded to each author<br>• The full name of each funder<br>• URL of each funder website<br>• Did the sponsors or funders play any role in the study design, data collection and analysis, decision to publish, or preparation of the manuscript?<br><br>Did you receive funding for this work?	
Please add funding details.<br>as follow-up to "**Financial Disclosure**<br><br>Enter a financial disclosure statement that describes the sources of funding for the work included in this submission. Review the submission guidelines for detailed requirements. View published research articles from *PLOS ONE* for specific examples.<br><br>This statement is required for submission and **will appear in the published article** if the submission is accepted. Please make sure it is accurate.<br><br>**Funded studies**<br>Enter a statement with the following details:<br>• Initials of the authors who received each award<br>• Grant numbers awarded to each author<br>• The full name of each funder<br>• URL of each funder website<br>• Did the sponsors or funders play any role in the study design, data collection and analysis, decision to publish, or preparation of the manuscript?	FM: National Institute of General Medical Sciences of the National Institutes of Health under Award Number R35GM146868. https://www.nigms.nih.gov/. The funders had no role in study design, data collection and analysis, decision to publish, or preparation of the manuscript.

Did you receive funding for this work?"	
Please select the country of your main research funder (please select carefully as in some cases this is used in fee calculation).<br>as follow-up to "**Financial Disclosure**<br><br>Enter a financial disclosure statement that describes the sources of funding for the work included in this submission. Review the submission guidelines for detailed requirements. View published research articles from *PLOS ONE* for specific examples.<br><br>This statement is required for submission and **will appear in the published article** if the submission is accepted. Please make sure it is accurate.<br><br>**Funded studies**<br>Enter a statement with the following details:<br>• Initials of the authors who received each award<br>• Grant numbers awarded to each author<br>• The full name of each funder<br>• URL of each funder website<br>• Did the sponsors or funders play any role in the study design, data collection and analysis, decision to publish, or preparation of the manuscript?<br><br>Did you receive funding for this work?"	UNITED STATES - US
**Competing Interests**<br><br>Use the instructions below to enter a competing interest statement for this submission. On behalf of all authors, disclose any competing interests that could be perceived to bias this work—acknowledging all financial support and any other relevant financial or non-financial competing interests.<br><br>This statement is required for submission	The authors have declared that no competing interests exist.

and **will appear in the published article** if the submission is accepted. Please make sure it is accurate and that any funding sources listed in your Funding Information later in the submission form are also declared in your Financial Disclosure statement.<br><br>View published research articles from *PLOS ONE* for specific examples.<br><br>**NO authors have competing interests**<br><br>Enter: *The authors have declared that no competing interests exist.*<br><br>**Authors with competing interests**<br><br>Enter competing interest details beginning with this statement:<br><br>*I have read the journal's policy and the authors of this manuscript have the following competing interests: [insert competing interests here]*<br><br>* typeset	
**Ethics Statement**<br><br>Enter an ethics statement for this submission. This statement is required if the study involved:<br><br>• Human participants<br>• Human specimens or tissue<br>• Vertebrate animals or cephalopods<br>• Vertebrate embryos or tissues<br>• Field research<br><br>Write "N/A" if the submission does not require an ethics statement.<br><br>General guidance is provided below. Consult the submission guidelines for detailed instructions. **Make sure that all information entered here is included in the Methods section of the manuscript.**	N/A

**Format for specific study types**<br><br>**Human Subject Research (involving human participants and/or tissue)**<br>• Give the name of the institutional review board or ethics committee that approved the study<br>• Include the approval number and/or a statement indicating approval of this research<br>• Indicate the form of consent obtained (written/oral) or the reason that consent was not obtained (e.g. the data were analyzed anonymously)<br><br>**Animal Research (involving vertebrate animals, embryos or tissues)**<br>• Provide the name of the Institutional Animal Care and Use Committee (IACUC) or other relevant ethics board that reviewed the study protocol, and indicate whether they approved this research or granted a formal waiver of ethical approval<br>• Include an approval number if one was obtained<br>• If the study involved *non-human primates*, add *additional details* about animal welfare and steps taken to ameliorate suffering<br>• If anesthesia, euthanasia, or any kind of animal sacrifice is part of the study, include briefly which substances and/or methods were applied<br><br>**Field Research**<br><br>Include the following details if this study involves the collection of plant, animal, or other materials from a natural setting:<br>• Field permit number<br>• Name of the institution or relevant body that granted permission	
**Data Availability**<br><br>Authors are required to make all data underlying the findings described fully available, without restriction, and from the time of publication. PLOS allows rare exceptions to address legal and ethical concerns. See the PLOS Data Policy and FAQ for detailed information.	Yes - all data are fully available without restriction

Powered by Editorial Manager® and ProduXion Manager® from Aries Systems Corporation

A Data Availability Statement describing where the data can be found is required at submission. Your answers to this question constitute the Data Availability Statement and **will be published in the article**, if accepted.<br><br>**Important:** Stating 'data available on request from the author' is not sufficient. If your data are only available upon request, select 'No' for the first question and explain your exceptional situation in the text box.<br><br>Do the authors confirm that all data underlying the findings described in their manuscript are fully available without restriction?	
**Describe where the data may be found in full sentences. If you are copying our sample text, replace any instances of XXX with the appropriate details.**<br><br>• If the data are **held or will be held in a public repository**, include URLs, accession numbers or DOIs. If this information will only be available after acceptance, indicate this by ticking the box below. For example: *All XXX files are available from the XXX database (accession number(s) XXX, XXX.)*.<br>• If the data are all contained **within the manuscript and/or Supporting Information files**, enter the following: *All relevant data are within the manuscript and its Supporting Information files.*<br>• If neither of these applies but you are able to provide **details of access elsewhere**, with or without limitations, please do so. For example:<br><br>*Data cannot be shared publicly because of [XXX]. Data are available from the XXX Institutional Data Access / Ethics Committee (contact via XXX) for researchers who meet the criteria for access to confidential data.*<br><br>*The data underlying the results presented in the study are available from (include the name of the third party*	All DGRP lines are available from the Bloomington Drosophila Stock Center (Bloomington, IN). All raw and processed RNA-Seq data are available at the NCBI Gene Expression Omnibus (GEO; https://www.ncbi.nlm.nih.gov/geo/) under accession number GSE117850. Phenotypic data are available at http://dgrp2.gnets.ncsu.edu/. The code used for the analyses is available at https://github.com/nklimko/dgrp-starve.

Powered by Editorial Manager® and ProduXion Manager® from Aries Systems Corporation

and contact information or URL).<br>• This text is appropriate if the data are owned by a third party and authors do not have permission to share the data.<br><br>* typeset	
Additional data availability information:	

Manuscript

Click here to access/download;Manuscript;dgrp_transcriptomic_pred_manuscr

Comparing statistical learning methods for complex trait prediction from gene expression

Noah Klimkowski Arango[1,2], Fabio Morgante[1,2*]

1 Center for Human Genetics, Clemson University, Greenwood, SC, USA
2 Department of Genetics and Biochemistry, Clemson University, Clemson, SC, USA

* fabiom@clemson.edu

Abstract

Accurate prediction of complex traits is an important task in quantitative genetics that has become increasingly relevant for personalized medicine. Genotypes have traditionally been used for trait prediction using a variety of methods such as mixed models, Bayesian methods, penalized regressions, dimension reductions, and machine learning methods. Recent studies have shown that gene expression levels can produce higher prediction accuracy than genotypes. However, only a few prediction methods were used in these studies. Thus, a comprehensive assessment of methods is needed to fully evaluate the potential of gene expression as a predictor of complex trait phenotypes. Here, we used data from the *Drosophila* Genetic Reference Panel (DGRP) to compare the ability of several existing statistical learning methods to predict starvation resistance from gene expression in the two sexes separately. The methods considered differ in assumptions about the distribution of gene effect sizes – ranging from models that assume that every gene affects the trait to more sparse models – and their ability to capture gene-gene interactions. We also used functional annotation (*i.e.*, Gene Ontology (GO)) as an external source of biological information to inform prediction models. The results show that differences in prediction accuracy between methods exist, although they are generally not large. Methods performing variable selection gave higher accuracy in females while methods assuming a more polygenic architecture performed better in males. Incorporating GO annotations further improved prediction accuracy for a few GO terms of biological significance. Biological significance extended to the genes underlying highly predictive GO terms with different genes emerging between sexes. Notably, the Insulin-like Receptor (*InR*) was prevalent across methods and sexes. Our results confirmed the potential of transcriptomic prediction and highlighted the importance of selecting appropriate methods and strategies in order to achieve accurate predictions.

Introduction

Predicting yet-to-be observed phenotypes for complex traits is an important task for many branches of quantitative genetics. Complex trait prediction was developed in agricultural breeding to select the best performing individuals for economically important traits such as milk yield in dairy cattle using estimated breeding values (EBVs). While EBVs have been traditionally computed using pedigree information, with the availability of genotyping arrays, EBVs have been replaced or supplemented with their genomic counterpart – genomic EBVs (GEBVs) [1, 2]. GEBVs are linear

combinations of the genotypes of the target individuals and the effect sizes of many genetic variants along the genome computed in a reference population. The same concept has later been applied to human genetics, especially in the context of precision medicine. Here the goal is to predict medically relevant phenotypes such as body mass index (BMI) or disease susceptibility using Polygenic Scores (PGSs) [3, 4]. While GEBVs and PGSs are technically the same, the different goals of these two quantities (*i.e.*, selection for GEBVs and prevention/monitoring for PGS) have important implications. We refer the readers to [2] for a comprehensive treatment of this topic.

The estimation of the effect sizes of genetic variants to be used for prediction can be done using a variety of methods. The most common methods have regression at their core, where the response variable is the phenotype of interest and the predictor variables are the genotypes [5]. Because the number of genetic variants is usually much larger than the sample size, methods that perform some regularization of the effect sizes are needed. These methods encompass dimension reduction methods (*e.g.*, principal components regression), penalized regression methods (*e.g.*, ridge regression), linear mixed models (*e.g.*, GBLUP), Bayesian methods (*e.g.*, BayesC), and machine learning (*e.g.*, random forest) [6–10]. These methods differ in the assumptions they make regarding the distribution of the effect sizes, with some methods performing only effect shrinkage and some methods performing variable selection as well [5]. Research focused on comparing several methods has shown that there is no single best method, with performance being affected by the genetic architecture of the trait of interest (*e.g.*, sparse vs dense), the biology of the species (*e.g.*, the extent of linkage disequilibrium), and assumptions of the method [11, 12].

Traditionally, genotype data has been used for complex trait prediction since they are easy and cost-effective to obtain. However, it is now possible to obtain multidimensional molecular data such as gene expression or metabolite levels at a reasonable cost. This has opened to the possibility of using these additional layers of data for complex trait prediction. Given that genetic information flows from DNA to RNA, to proteins, to metabolites to affect phenotypes [13], using these intermediate layers of data could improve prediction accuracy for at least some traits. In addition, to being biologically 'closer' to phenotypes, gene expression levels, protein levels, and metabolite levels can be thought of as endophenotypes, which are affected by environmental conditions as well. Thus, endophenotypes could capture environmental and gene-by-environment effects [14]. Recent work has shown that using additional omic data types can result in more accurate predictions [14–19]. In particular, using transcriptomic data has shown good promise. For example, Wheeler *et al.* used lymphoblastoid cell line data to show that gene expression levels provided much higher accuracy than genotypes at predicting intrinsic growth rate [15]. In a similar fashion, Morgante *et al.* found that prediction accuracy of starvation resistance in *Drosophila melanogaster* was higher when using gene expression levels than when using genotypes [17].

While these studies have shown the potential of gene expression as a predictor of complex phenotypes, only a few statistical methods were used, with most studies using linear mixed models. However, as discussed above, studies using genotypes have found that prediction accuracy can vary substantially depending on the method used. Thus, in this study we sought to compare several common methods spanning dimensionality reductions, penalized linear regressions, Bayesian linear regressions, linear mixed models, and machine learning in their ability to predict starvation resistance from gene expression levels using data from the *Drosophila* Genetic Reference Panel [20].

Materials and Methods

Data Processing

The Drosophila melanogaster Genetic Reference Panel (DGRP) is a collection of over 200 inbred lines derived from a natural population that have full genome sequences and phenotypic measurements for several traits [21]. Additionally, prior work from [22] obtained full transcriptome profiles by RNA sequencing for a total of 200 DGRP lines. Following the filtering steps described in [17], we ended up with 11,338 genetically variable and highly expressed genes in females and 13,575 in males. Among the many traits available for the DGRP, in this work we used starvation resistance as a model trait because it can be predicted with decent accuracy with the small sample size available [17]. In particular, line means for 198 lines that have both transcriptome profiles and phenotypic measurements, adjusted for the effect of *Wolbachia* infection and major inversions [20] were used for all the analyses.

Transcriptomic Prediction

The methods used follow the general multiple regression model:

$$\mathbf{y} = \mathbf{X}\boldsymbol{\beta} + \mathbf{e} \tag{1}$$

where $\mathbf{y}$ is a n-vector of phenotypes, $\mathbf{X}$ is a $n \times m$ matrix of expression levels for m genes, $\boldsymbol{\beta}$ is a m-vector of effect sizes, and $\mathbf{e}$ is a n-vector of residuals. We assume that the columns of $\mathbf{X}$ and $\mathbf{y}$ have been centered to mean 0.

Principal Component Regression (PCR)

PCR [23] uses Principal Component Analysis [24] to reduce the dimensionality of the predictor matrix, $\mathbf{X}$, by selecting a set of k orthogonal components that are linear combinations of the original predictors and maximize their variance. Then, the $n \times k$ matrix of principal components, $\mathbf{T}$, is used in place of $\mathbf{X}$ in equation 1 [25]. We used the algorithm implemented in the R package pls v. 2.8-2 ([26]) with default parameters. We used 5-fold cross validation in the training set to select the number of principal components to be used for prediction in the test set.

Partial Least Squares Regression (PLSR)

Like PCR, PLSR [27] also reduces the dimensionality of the predictor matrix, $\mathbf{X}$. However, this is achieved via a simultaneous decomposition of $\mathbf{X}$ and $\mathbf{y}$ and selecting a set of k components that maximizes the covariance between $\mathbf{X}$ and $\mathbf{y}$. This addresses a limitation of PCR that the components that best "explain" $\mathbf{X}$ may not necessarily be the most relevant to $\mathbf{Y}$. Then, the $n \times k$ matrix of latent vectors, $\mathbf{T}$, is used in place of $\mathbf{X}$ in equation 1 [28]. We used the algorithm implemented in the R package pls v. 2.8-2 ([26]), setting the method to 'widekernelpls' which is suitable for the wide ($m >> n$) matrix of gene expression, and other default parameters. We used 5-fold cross validation in the training set to select the number of latent vectors to be used for prediction in the test set.

Ridge Regression (RR)

Ridge Regression [6] is a penalized linear regression method that uses an ℓ_2 penalty to achieve shrinkage of the estimates of the effect sizes. The amount of shrinkage is

determined by a tuning parameter, λ, such that large values of λ result in more shrinkage. We used the algorithm implemented in the R package glmnet v. 4.1-8 ([29]). We used 5-fold cross validation to select λ. All other parameters were left as their default values.

Least Absolute Shrinkage Selector Operator (LASSO)

LASSO [30] is a penalized linear regression method that uses an ℓ_1 penalty to perform both variable selection (by setting some effects to be exactly 0) and shrinkage of the estimates of the effect sizes. The amount of shrinkage and variable selection is determined by a tuning parameter, λ, such that large values of λ result in more shrinkage. We used the algorithm implemented in the R package glmnet v. 4.1-8 ([29]) was fit. We used 5-fold cross validation to select λ. All other parameters were left as their default values.

BayesC

BayesC [31] is a Bayesian linear regression method that imposes a spike-and-slab prior on the effect sizes:

$$\boldsymbol{\beta}_j \sim \pi N(0, \sigma^2_\beta) + (1-\pi)\delta_0 \qquad (2)$$

where π is the probability that the effect of jth variable comes from a normal distribution with mean 0 and variance σ^2_β, and δ_0 is a point-mass at 0. In this way, both variable selection and effect shrinkage are achieved. In the R package BGLR v. 1.1.0 implementation, [32], the posterior distribution of the effect sizes and some model parameters are computed using Markov Chain Monte Carlo (MCMC) methods. We ran the algorithm for 130,000 iterations, with the first 30,000 iterations discarded as burn-in and retaining every 50th sample. We assessed convergence through visual inspection of the trace plots. The expected proportion of variance by the predictors, R^2, was set to 0.8 to in line with the broad sense heritability of starvation resistance [33].

Variational Bayesian Variable Selection (VARBVS)

VARBVS is a Bayesian linear regression method that imposes the same spike-and-slab prior as BayesC. However, posterior computations are done using Variational Inference rather than MCMC, which is more computationally efficient [34]. The algorithm implemented in the R package varbvs v. 2.6-10 ([34]) was fit with default parameters.

Multiple Regression with Adaptive Shrinkage (MR.ASH)

MR.ASH is a Bayesian linear regression method that imposes a scale mixture-of-normals prior on the effect sizes:

$$\boldsymbol{\beta}_j|\pi, \boldsymbol{\sigma^2} \sim \pi_0\delta_0 + \sum_{k=1}^{K} \pi_k N(0, \sigma^2_k) \qquad (3)$$

for a fixed grid of variances, $\boldsymbol{\sigma^2}$. Thus, like BayesC and VARBVS, MR.ASH performs both variable selection and effect shrinkage, but is able to model more complex distributions of the effect sizes owing to a more flexible prior. MR.ASH uses a Variational Empirical Bayes approach to estimate the prior (*i.e.*, the mixture weights, π) from the data and compute the posterior distribution of the effect sizes [35]. The algorithm implemented in the R package mr.ash.alpha v. 0.1-43 ([36]) was fit with default parameters, but used the effect size estimates from LASSO to initialize MR.ASH.

Transcriptomic Best Linear Unbiased Predictor (TBLUP)

TBLUP [37] is a linear mixed model that aggregates the effects of all the genes into a single random effect. Let $\mathbf{t} = \sum_{j=1}^{m} \mathbf{w}_j \boldsymbol{\beta}_j = \mathbf{W}\boldsymbol{\beta}$, where $\mathbf{w}_j$ is a standardized version of $\mathbf{x}_j$ to have variance 1, then:

$$\mathbf{y} = \mathbf{t} + \mathbf{e} \tag{4}$$

where $\mathbf{t}$ is a n-vector of transcriptomic effects, $\mathbf{t} \sim N(0, \mathbf{T}\sigma^2_{\mathbf{t}})$, and $\mathbf{T} = \dfrac{\mathbf{W}\mathbf{W}^{\mathsf{T}}}{m}$ is the Transcriptomic Relationship Matrix (TRM). TBLUP was implemented by using theR package BGLR v. 1.1.0 ([32]). BGLR uses a Bayesian approach to estimate the transcriptomic and residual variance components. We ran the algorithm for 85,000 iterations, with the first 10,000 iterations discarded as burn-in and retaining every 50th sample. We assessed convergence through visual inspection of the trace plots. The expected proportion of variance, R^2, was set to 0.8. All other parameters were left as their default values.

Please provide reference or reason to this criterion

All the previous methods assume that no gene-gene interactions affect starvation resistance. Thus, we decided to add some more flexible machine learning methods to the comparison, which are also able to capture interaction effects.

Random Forest (RF)

Random Forest is a machine learning method whereby a collection of decision trees are grown, each on a different bootstrap sample of the predictor data [10]. This method has been used to identify gene-gene interactions successfully [38]. The model is given by:

$$\mathbf{y} = \sum_{s=1}^{S} c_s h_s(\mathbf{y}; \mathbf{X}) \tag{5}$$

where S is the number of decision trees, c_s is a shrinkage factor that averages the trees, $h_s(\mathbf{y}; \mathbf{X})$ is a decision tree that is grown using only a subset of predictors at each nodes [10]. The algorithm implemented in the R package partykit v. 1.2-20 ([39]) was fit with 1000 trees and default parameters.

Neural Network (NN)

Artificial Neural Networks are a type of machine learning method that use layers of nodes, or neurons, to process data similar to how the human brain works. Neural networks are built using input layers, hidden layers, and output layers. Input layers receive data while output layers calculate final results. Hidden layers are able to modify inputs from previous layers to discover trends or patterns within data [40]. Network assembly is tailored to individual problems, as networks can vary by hidden layer count, neuron count per layer, and activation function per layer. In our model, weights for a single layer of hidden neurons were estimated using resilient backpropagation [41]. Neural networks use nonlinear activation functions to determine whether neurons in hidden layers should be activated based on their inputs. This feature can be used to model gene interactions [42]. The general model for an artificial neural network is given by

$$y = a\left(\sum_{i=0}^{m} \mathbf{w}_i \mathbf{x}_i\right)$$

where y is the phenotype of a given line, a is an activation function, m is the number of inputs, $\mathbf{w}$ is the vector of weights, $\mathbf{x}$ is a vector of gene expression, and w_0 is the bias or intercept of the model. The neural network implemented through the R package

neuralnet v1.44.2 ([43]) used default parameters and a custom neuron structure. Neuron count selection is a fundamental problem in constructing networks [44]. We specified a hidden layer size of 1,000 neurons.

Gene Ontology Informed Transcriptomic Prediction

While some of the methods above try to enrich the prediction model for genes that are particularly predictive of the trait by performing internal variable selection, this procedure becomes difficult with small sample size such as in the DGRP. Informing prediction models with functional annotation has been shown to be effective at disentangling signal from noise and improve accuracy in complex trait prediction [17, 45–47]. Edwards *et al.* (2016) and Morgante *et al.* (2020) used Gene Ontology (GO) [48] to improve prediction accuracy for three complex traits in *Drosophila* [17, 45]. However, these applications only used BLUP-type models to include GO information. Here, we tested a few additional methods described below. For each sex, we selected GO terms that included at least five genes present in the DGRP expression data as done in previous work [17]. This procedure resulted in 2,628 terms for females and 2,580 terms for males being retained for further analysis. For all methods, GO-informed models were fit with one GO term at a time for all GO terms specified for each sex.

Sparse Group LASSO

Sparse group LASSO [49] is a penalized regression method that uses a combination of the ℓ_1 penalty and a group LASSO penalty [50]. The Group LASSO [51] applies variable selection on entire groups of predictors, while the ℓ_1 penalty achieves effect shrinkage and variable selection at the individual variable level. The strength of the penalties is determined by a tuning parameter, λ, such that larger values of λ result in more shrinkage/selection. In our application, one group included all the genes in the selected GO term and the other group included all the remaining genes. We used the Sparse Group LASSO implementation in the R package sparseGL v1.0.2 [52] with default parameters.

GO-BayesC

GO-BayesC is a Bayesian linear regression method that imposes independent spike-and-slab priors on effect sizes of genes grouped by GO term association. Let

$$\mathbf{y} = \mathbf{X}_{GO}\boldsymbol{\beta}_{GO} + \mathbf{X}_{notGO}\boldsymbol{\beta}_{notGO} + \mathbf{e} \tag{6}$$

where $\mathbf{X}_{GO}$ is the subset of $\mathbf{X}$ containing the genes associated with the selected GO term, $\boldsymbol{\beta}_{GO}$ is the vector of effects of the genes in the selected GO term, $\mathbf{X}_{notGO}$ is the subset of $\mathbf{X}$ containing all other genes, and β_{notGO} the vector of effects of all other genes. $\beta_{GO,j}$ and $\boldsymbol{\beta}_{notGO,j}$ are assigned separate spike-and-slab prior distributions as in equation 2. This method uses the same algorithm from the R package BGLR [32]. We ran the algorithm for 130,000 iterations, with the first 30,000 iterations discarded as burn-in and retaining every 50th sample. We assessed convergence through visual inspection of the trace plots. The expected proportion of variance by all the predictors, R^2, was set to 0.8.

GO-TBLUP

GO-TBLUP is an extension of TBLUP that includes two random effects, one associated with genes in the selected GO term and one associated with all the other genes:

$$\mathbf{y} = \mathbf{t}_{GO} + \mathbf{t}_{notGO} + \mathbf{e} \tag{7}$$

where $\mathbf{t}_{GO}$ is a n-vector of transcriptomic effects associated with genes in the GO term, $\mathbf{t}_{GO} \sim N(0, \mathbf{T}_{GO}\sigma^2_{\mathbf{t}_{GO}})$, $\mathbf{T}_{GO} = \frac{\mathbf{W}_{GO}\mathbf{W}_{GO}^\intercal}{m}$, $\mathbf{W}_{GO}$ is the subset of $\mathbf{W}$ containing the genes associated with the selected GO term, $\mathbf{t}_{notGO}$ is a n-vector of transcriptomic effects associated with all other genes, $\mathbf{t}_{notGO} \sim N(0, \mathbf{T}_{notGO}\sigma^2_{\mathbf{t}_{notGO}})$, $\mathbf{T}_{notGO} = \frac{\mathbf{W}_{notGO}\mathbf{W}_{notGO}^\intercal}{m}$, and $\mathbf{W}_{notGO}$ is the subset of $\mathbf{W}$ containing all other genes. GO-TBLUP was implemented by using the R package BGLR v. 1.1.0 ([32]). BGLR uses a Bayesian approach to estimate the transcriptomic and residual variance components. We ran the algorithm for 85,000 iterations, with the first 10,000 iterations discarded as burn-in and retaining every 50th sample. We assessed convergence through visual inspection of the trace plots. The expected proportion of variance, R^2, was set to 0.8. All other parameters were left as their default values.

Evaluation Scheme

We fitted each method to 90% of the data (*i.e.*, training set) to estimate the model parameters and used them to predict phenotypes for the remaining 10% of the data (*i.e.*, test set). Prediction accuracy was measured as the correlation coefficient between actual and predicted phenotypes. We repeated this procedure for 25 random training-test splits and used the average correlation across splits as our final metric to assess prediction accuracy.

Results

Transcriptomic Prediction

We first fitted a few widely used regression methods and compared their prediction accuracy. The results are shown in Fig 1 and S1 Table.. Overall, prediction accuracy was moderately low, especially considering that the analyses were based on lines means of many individual flies, resulting in the majority of the phenotypic variance being genetic [17]. However, the results show that differences in prediction accuracy between methods exist, both within sex and across sexes. In males, we found that TBLUP (mean $r = 0.455 \pm 0.035$) and Ridge Regression ($r = 0.455 \pm 0.034$) provided the highest accuracy, with BayesC (mean $r = 0.448 \pm 0.033$), PLSR (mean $r = 0.442 \pm 0.034$), and PCR (mean $r = 0.430 \pm 0.039$) being competitive. On the other hand, Neural Network provided the lowest prediction accuracy for males ($r = 0.143 \pm 0.079$). In females, we observed more marked differences in prediction accuracy across methods. Methods that perform variable selection – *i.e.*, VARBVS, MR.ASH, and LASSO – tended to perform better than the other methods, with VARBVS (mean $r = 0.503 \pm 0.031$) providing the highest accuracy. Neural Network provided the lowest prediction accuracy in females ($r = 0.064 \pm 0.051$) as well.

Fig 1. Prediction accuracy of 25 replicates in A) females and B) males for all standard methods. Methods are colored by family. The mean correlation coefficient is denoted by diamonds. Outliers are denoted by circles.

Gene Ontology Informed Transcriptomic Prediction

It has been shown previously that informing prediction models with functional information can help achieve higher prediction accuracy [17, 45–47]. Thus, we also tested methods that could include external information. In this work, we focused on GO annotation and extension of BayesC and TBLUP, namely GO-BayesC and GO-TBLUP. The results are summarized in Fig 2 and S2 Table.. We also sought to use the Sparse Group LASSO. However, in our initial testing, the prediction accuracies provided by that method were nearly identical for all GO terms tested (S1 Fig.). This pattern was also seen for GO terms found to be highly predictive in GO-BayesC and GO-TBLUP. Thus, we decided not to assess Sparse Group Lasso further.

Fig 2. Prediction accuracy using GO-BayesC in females (A) and males (B). Prediction accuracy using GO-TBLUP in females (C) and males (D). Each dot represents the mean correlation between true and predicted phenotypes (r) across 25 replicates for a GO term. The solid line indicates the mean r from the respective standard method (*i.e.*, BayesC and TBLUP). The dashed black line represents the 99th percentile of terms ranked by prediction accuracy.

We found that GO-BayesC and GO-TBLUP provided accuracies that were similar to or lower than the respective standard model (*i.e.*, BayesC and TBLUP) for the majority of GO terms in both sexes. However, some GO terms seemed to be particularly predictive of the trait, yielding accuracies that are substantially higher than the standard models. The accuracies provided by GO-BayesC and GO-TBLUP generally agreed ($r = 0.836$), as shown in Fig 3.

Fig 3. Plot of prediction accuracy for all GO terms using GO-BayesC(x-axis) against GO-TBLUP (y-axis) for A) females and B) males. The black line represents the line of best fit for each panel.

In females, four of the five most predictive GO terms are shared between GO-BayesC and GO-TBLUP. GO terms GO:0017056 (GO-BayesC $r = 0.477 \pm 0.034$, GO-TBLUP $r = 0.457 \pm 0.041$) and GO:0006606 (GO-BayesC $r = 0464. \pm 0.039$, GO-TBLUP $r = 0.434 \pm 0.044$) are both related to nuclear import by function and structure, respectively. Lee *et al.* has demonstrated starvation resistance inducing nuclear pore degradation in yeast [53]. The other two GO terms GO:0055088 (GO-BayesC $r = 0.446 \pm 0.040$, GO-TBLUP $r = 0.460 \pm 0.036$) and GO:0045819 (GO-BayesC $r = 0.451 \pm 0.038$, GO-TBLUP $r = 0.427 \pm 0.042$) are related to macromolecule metabolism in lipids and carbohydrates, respectively. GO:0055088 has been implicated in starvation resistance using Korean rockfish [54]. GO:0017056 was the most predictive term for GO-BayesC ($r = 0.477 \pm 0.034$) and GO-TBLUP ($r = 0.457 \pm 0.041$). However, some differences exist. For example, GO:0016042, which is involved with lipid catabolism, was found to be highly predictive by GO-BayesC ($r = 0.447 \pm 0.038$), while GO:0008586, which is involved with wing vein morphogenesis, was highly predictive in GO-TBLUP ($r = 0.424 \pm 0.039$).

In males, two of the five most predictive GO terms are shared between methods. GO:0042593 (GO-BayesC $r = 0539. \pm 0.032$, GO-TBLUP $r = 0.520 \pm 0.035$) is involved in glucose homeostasis, while GO:0035003 (GO-BayesC $r = 0.526 \pm 0.036$, GO-TBLUP $r = 0.512 \pm 0.030$) is involved in the subapical complex. which is a key component of the intestinal epithelial tissue. As part of epithelial tissue, the subapical complex is involved with nutrient acquisition in the intestines as part of the barrier between host cells and the gut microbiome. [55] Four of the top five GO terms found from either method are implicated in cellular growth and development. In GO-BayesC, GO:0042461

($r = 0.530 \pm 0.036$) is involved in photoreceptor cells, GO:0001738 ($r = 0.530 \pm 0.036$) is involved in epithelial tissue and GO:0045186 ($r = 0.529 \pm 0.037$) is involved in the assembly of the zonula adherens. GO:0007485, which is involved in genital disc formation, was highly predictive in GO-TBLUP($r = 0.520 \pm 0.030$). Multiple studies have found connections between cell size regulation and overall body size with starvation resistance [56–58]. The top GO term for TBLUP in males GO:0035008($r = 0.526 \pm 0.026$), is involved in the positive regulation of the melanization defense response. The biological connection of this process to starvation resistance is unclear, as this response increases oxidative stress in wounds to prevent infection [59].

Gene Analysis

Given that many of the most predictive GO terms were biologically relevant to starvation resistance, we investigated whether any particular genes were included in such terms. To do so, we selected the 1% most predictive GO terms for each method and sex (26 and 25 GO terms for females and males, respectively). For each prediction method and sex combination, we counted how many times each gene was found across these GO terms. We then examined the distribution of the count (S2 Fig.) and decided to focus only on the most frequently occurring genes. This resulted in selecting genes appearing in 5 or more GO terms for GO-TBLUP in females and genes appearing in 4 or more GO terms for all other method and sex combinations. These results are summarized in Table 1 and S3 Table..

Table 1. Top overlapping genes across the 1% most predictive GO terms for each method and sex combination.

GO-BayesC Female		GO-TBLUP Female		GO-BayesC Male		GO-TBLUP Male	
**Gene**	**Count**	**Gene**	**Count**	**Gene**	**Count**	**Gene**	**Count**
AkhR	9	*Egfr*	8	*sdt*	13	*sdt*	8
mbo	6	*Akt1*	6	*aPKC*	9	*aPKC*	7
InR	6	AkhR	6	*par-6*	8	*PDZ-GEF*	6
Nup54	5	*InR*	6	*Patj*	8	*par-6*	6
Akh	5	*Sik3*	5	*scrib*	5	*Patj*	5
Nup154	4	*emc*	5	*baz*	5	*crb*	5
Nup93-1	4	*put*	5	*crb*	5	*Ilp2*	4
Nup205	4	*babo*	5	*Moe*	5	*Desat1*	4
Nup93-2	4	*Pdk1*	5	*PDZ-GEF*	5	*InR*	4
Nup98-96	4	*Ack*	5	*AkhR*	4	*Ras85D*	4
Nup153	4	*Pkc98E*	5	*Ilp2*	4		
Akt1	4	*CG3216*	5	*InR*	4		
Pi3K92E	4	*CG31183*	5	*dlg1*	4		
Egfr	4	*Erk7*	5	*shg*	4		
Erk7	4	*hpo*	5				
		hep	5				

Genes appearing in all four setups are highlighted in green. Genes appearing in both methods for females are highlighted in red. Genes appearing for both methods in males are highlighted in blue.

For both sexes, the results show that some overlapping genes were found by both GO-BayesC and GO-TBLUP while some genes were picked up by only one method. One trend that emerged across all setups a significant enrichment (Fisher's Exact Test $P < 0.001$) of protein kinases among the top genes.

In females, GO-BayesC and GO-TBLUP found 5 genes in common out of the top 1%

of GO terms. These five genes are related to insulin signaling and lipid metabolism. Insulin signaling is involved in cell growth, feeding, carbohydrate metabolism, and many other traits critical for survival [60]. The insulin-like receptor (*InR*) is crucial for insulin signaling in carbohydrate metabolism. The adipokinetic hormone receptor (*AkhR*) is responsible for both carbohydrate and lipid metabolism signals. *AkhR* was shown to coexpress with *InR* on starvation-induced hyperactivity [61]. *Akt1* is the core kinase subunit of the insulin growth factor pathway and has been implicated in starvation resistance in a cancer study [62]. Aside from insulin signaling, the top two genes shared between methods have been implicated in starvation resistance. Epidermal growth factor receptor (*Egfr*) is important for normal cell growth, while overexpression of the receptor is a common route for cancer development [63]. Sevelda *et al.* showed that *Egfr* increased starvation resistance through interactions with *Akt1* [64]. *Erk7* is an extracellular kinase involved in the secretory system. *Erk7* downregulates secretion by triggering the destruction of endoplasmic reticulum exit sites under starvation conditions [65].

Most of the genes found by GO-BayesC only are from the nucleoporin family (*mbo, Nup53, Nup54, Nup93-1, Nup93-2, Nup98-96, Nup153, Nup205*). Nucleoporin degradation can occur under starvation conditions to prevent the export of macromolecules [53]. The remaining genes are tangential to the *InR* signaling pathway. The adipokinetic hormone (*Akh*) makes a complex with its receptor *AkhR* previously described as part of the *InR* signaling pathway. Downstream in the pathway, a signal propagating kinase *Pi3K92E* was also found by GO-BayesC. Eleven distinct genes found by GO-TBLUP only are involved in various complexes and pathways. Three of these genes are related to the Hippo, or Salvador-Warts-Hippo, signaling pathway [66] — the core signaling kinase hippo (*hpo*), a kinase further downstream (*ack*), and *Sik3*. *Sik3* is involved in balancing NADPH/NADP+, a set of key components for oxidative stress mitigation and cellular signaling [67]. *Sik3* is also part of the *InR* signaling pathway that negatively regulates Hippo signaling. Another top gene (*pdk1*) is part of the *InR* signaling pathway and is responsible for inhibiting apoptosis during embryonic development. In flies, activin signaling responds to carbohydrate levels in the gut by increasing carbohydrase expression [68]. Additionally, activin signaling increases starvation resistance for neuronal cells [69]. The products of two genes, *put* and *babo*, form a complex with an activin-like ligand to initiate the activin signaling pathway [70]. Hemipterous (*hep*) is a kinase involved in imaginal disc formation and cell proliferation. A study found that *hep* loss-of-function mutant flies have reduced energy stores and lower starvation resistance. [71] *hep* has also been implicated in obesity and insulin resistance by activating the JNK signaling pathway [72].

In males, GO-BayesC and GO-TBLUP found eight genes in common out of the top 1% of GO terms. The two major categories that emerge from these genes are carbohydrate metabolism and cell polarization. For carbohydrate metabolism, the insulin-like receptor (*InR*) and an insulin-like peptide (*Ilp2*) are key components of *InR* signaling. *InR* is the only gene that was found by both GO methods in both sexes. The remaining genes are all related to cell polarization. The Crumbs complex has three genes (*crb, sdt, Patj*), while the PAR complex has two genes (*aPKC* and *par-6*) from both methods. Both complexes are highly conserved regulators of apico-basal cell formation [73]. The PAR complex negatively regulates the *InR* signaling pathway [74]. Outside of these complexes, *PDZ-GEF* is a guanine exchange factor involved in epithelial cell polarization through a separate mechanism [75].

Six genes were found by GO-BayesC only in the top 1% of GO terms from males. Three genes are related to the PAR complex. Bazooka(*baz*) is a core component of the PAR complex while two genes, *scrib* and *dlg1*, form a conserved complex with *lgl* that regulates cell migration with the PAR complex [76]. The product of *Moe* competes with

PAR complex component *aPKC* to regulate the Crumbs complex [77]. *AkhR* was found uniquely by GO-BayesC. As previously described, this gene is involved in carbohydrate and lipid metabolism signaling processes [61]. Another gene, shotgun(*shg*), is downstream in the *Egfr* signaling pathway [78]. As previously described, *Egfr* promotes starvation resistance through *Akt1* interactions [64]. Only two genes were found uniquely by GO-TBLUP in males. One is a desaturase (*Desat1*) that synthesizes fatty acid molecules. *Desat1* has been shown to induce cell autophagy under starvation conditions [79]. The other, *Ras85D*, is an oncogenic cell growth promoter. Downregulating *Ras85D* has been shown to improve starvation resistance by limiting growth signals [80].

Discussion

In this study, we evaluated ten statistical methods on their ability to predict starvation resistance, a well-documented quantitative complex trait [21], using transcriptomic data [22]. As expected, we found differences in the prediction accuracy provided by methods tested, both within sex and between sexes. While most methods were somewhat predictive, neural networks provided minimal prediction accuracy for both sexes. This is in agreement with previous work showing the importance of feature selection prior to model fitting in the $n << p$ regime for neural networks to perform well [11]. However, here we wanted to focus on out-of-the-box performance of the different methods. The most predictive methods in females, VARBVS and MR.ASH, are both Bayesian regression methods that perform effect shrinkage and variable selection. These methods allow for the underlying genetic architecture to be sparse, suggesting that not all genes affect starvation resistance. In contrast, the most predictive methods in males, TBLUP and ridge regression, only perform effect shrinkage, which suggests that the genetic architecture of starvation resistance is denser in males. The difference in best performing methods between sexes is not surprising because starvation resistance is a known sexually dimorphic trait with different genetic architectures between sexes [33, 81]. This difference highlights the importance of choosing methods with assumptions that match the complex trait under investigation. On the other hand, prediction analysis can also provide some hypothesis about the genetic architecture of the trait analyzed, to be followed up with more specific experiments.

Previous studies have shown that including functional annotation information into prediction models can help improve predictions [17, 45–47]. Thus, we selected two methods that allow the incorporation of additional information, BayesC and TBLUP, and annotated them with Gene Ontology (GO) information. We showed that a small number of biologically relevant GO terms achieved substantially higher prediction accuracies for GO-BayesC and GO-TBLUP than the standard BayesC and TBLUP. While the correlation between prediction accuracies for each GO between GO-BayesC and GO-TBLUP was high for both sexes ($r = 0.84$), differences in method characteristics resulted in different top GO terms between methods, especially in males. The most predictive GO terms for both sexes shared genes such as *InR* and *AkhR* that are involved in carbohydrate and/or lipid metabolism. Both genes have been associated to starvation resistance in previous studies [60, 61]. However, many of the genes shared by the most predictive GO terms in the two sexes were different (*e.g.*, *Egfr* in females and *sdt* in males). These findings also suggest that both methods may be able to highlight sex-shared and sex-specific genes of interest for traits with unknown genetic architectures. Overall, our findings suggest that additional information from GO terms may help disentangle signal from noise to improve prediction accuracy and our understanding of the complex trait of interest.

In conclusion, we found that differences in prediction performance between methods

exists and depend on the assumptions made by the model relative to the trait of interest. We also confirmed that external information such as GO term annotation can improve prediction accuracy for biologically relevant data. However, there are a number of limitations and considerations to address. First, the data is limited by the small number of available DGRP lines. Each transcriptome containing over 11,000 genes results in a $n << p$ problem that makes estimation of the relevant parameters problematic. We hypothesize that all methods would improve by increasing the sample size of the DGRP. Second, the linear regressions performed by most methods used here do not account for non-linear, or epistatic, interaction effects between genes and may perform poorly for complex traits with epistatic effects. Third, the gene expression data were obtained from whole flies under standard conditions. Higher prediction accuracy may be achieved if gene expression were measured under the same starvation conditions used to score starvation resistance, and for relevant tissues. Despite these limitations, we showed that gene expression data coupled with appropriate model selection can be effective for complex trait prediction.

Supporting information

S1 Fig. Violin plot comparison of Sparse Group Lasso results for top GO terms from GO-BayesC/GO-TBLUP along with randomly selected GO terms in females.

S2 Fig. Distribution of number of overlapping genes for top 1% of GO terms for GO-BayesC and GO-TBLUP in females and males. The selection cutoff is marked by the vertical bar.

S1 Table. Mean prediction accuracy and standard error for all methods in females and males.

S2 Table. Mean prediction accuracy and standard error for each GO term in GO-BayesC and GO-TBLUP for females and males.

S3 Table. Genes in the top 1% of GO terms for GO-BayesC and GO-TBLUP in females and males ordered by gene count.

Data and code availability

All DGRP lines are available from the Bloomington Drosophila Stock Center (Bloomington, IN). All raw and processed RNA-Seq data are available at the NCBI Gene Expression Omnibus (GEO; `https://www.ncbi.nlm.nih.gov/geo/`) under accession number GSE117850. Phenotypic data are available at `http://dgrp2.gnets.ncsu.edu/`. The code used for the analyses is available at `https://github.com/nklimko/dgrp-starve`.

Acknowledgments

We thank Trudy Mackay for helpful comments on an earlier version of this manuscript, and Liangjiang Wang for suggestions about the neural network analyses. Research reported in this publication was in part supported by the National Institute of General Medical Sciences of the National Institutes of Health under Award Number

R35GM146868 to FM. The content is solely the responsibility of the authors and does not necessarily represent the official views of the National Institutes of Health.

References

1. Meuwissen TH, Hayes BJ, Goddard M. Prediction of total genetic value using genome-wide dense marker maps. genetics. 2001;157(4):1819–1829.

2. Wray NR, Kemper KE, Hayes BJ, Goddard ME, Visscher PM. Complex trait prediction from genome data: contrasting EBV in livestock to PRS in humans: genomic prediction. Genetics. 2019;211(4):1131–1141.

3. Purcell SM, Wray NR, Stone JL, Visscher PM, O'Donovan MC, Sullivan PF, et al. Common polygenic variation contributes to risk of schizophrenia and bipolar disorder. Nature. 2009;460(7256):748–752.

4. Lewis CM, Vassos E. Polygenic risk scores: from research tools to clinical instruments. Genome medicine. 2020;12(1):44.

5. de Los Campos G, Hickey JM, Pong-Wong R, Daetwyler HD, Calus MP. Whole-genome regression and prediction methods applied to plant and animal breeding. Genetics. 2013;193(2):327–345.

6. Hoerl AE, Kennard RW. Ridge Regression: Biased Estimation for Nonorthogonal Problems. Technometrics. 1970;12(1):55–67. doi:10.1080/00401706.1970.10488634.

7. de Los Campos G, Vazquez AI, Fernando R, Klimentidis YC, Sorensen D. Prediction of complex human traits using the genomic best linear unbiased predictor. PLoS genetics. 2013;9(7):e1003608.

8. Habier D, Fernando RL, Kizilkaya K, Garrick DJ. Extension of the Bayesian alphabet for genomic selection. BMC bioinformatics. 2011;12:1–12.

9. Massy WF. Principal components regression in exploratory statistical research. Journal of the American Statistical Association. 1965;60(309):234–256.

10. Breiman L. Random Forests. Machine Learning. 2001;45(12):5–32.

11. Azodi CB, Bolger E, McCarren A, Roantree M, de Los Campos G, Shiu SH. Benchmarking parametric and machine learning models for genomic prediction of complex traits. G3: Genes, Genomes, Genetics. 2019;9(11):3691–3702.

12. Ma Y, Zhou X. Genetic prediction of complex traits with polygenic scores: a statistical review. Trends in Genetics. 2021;37(11):995–1011.

13. Civelek M, Lusis AJ. Systems genetics approaches to understand complex traits. Nature Reviews Genetics. 2014;15(1):34–48.

14. Azodi CB, Pardo J, VanBuren R, de Los Campos G, Shiu SH. Transcriptome-based prediction of complex traits in maize. The Plant Cell. 2020;32(1):139–151.

15. Wheeler HE, Aquino-Michaels K, Gamazon ER, Trubetskoy VV, Dolan ME, Huang RS, et al. Poly-omic prediction of complex traits: OmicKriging. Genetic epidemiology. 2014;38(5):402–415.

16. Guo Z, Magwire MM, Basten CJ, Xu Z, Wang D. Evaluation of the utility of gene expression and metabolic information for genomic prediction in maize. Theoretical and applied genetics. 2016;129:2413–2427.

17. Morgante F, Huang W, Sørensen P, Maltecca C, Mackay TF. Leveraging multiple layers of data to predict drosophila complex traits. G3: Genes, Genomes, Genetics. 2020;10(12):4599–4613.

18. Zhou S, Morgante F, Geisz MS, Ma J, Anholt RR, Mackay TF. Systems genetics of the Drosophila metabolome. Genome Research. 2020;30(3):392–405.

19. Rohde PD, Kristensen TN, Sarup P, Muñoz J, Malmendal A. Prediction of complex phenotypes using the Drosophila melanogaster metabolome. Heredity. 2021;126(5):717–732.

20. Huang W, Massouras A, Inoue Y, Peiffer J, Ràmia M, Tarone AM, et al. Natural variation in genome architecture among 205 Drosophila melanogaster Genetic Reference Panel lines. Genome research. 2014;24(7):1193–1208.

21. Mackay TF, Richards S, Stone EA, Barbadilla A, Ayroles JF, Zhu D, et al. The Drosophila melanogaster genetic reference panel. Nature. 2012;482(7384):173–178.

22. Everett LJ, Huang W, Zhou S, Carbone MA, Lyman RF, Arya GH, et al. Gene expression networks in the Drosophila genetic reference panel. Genome research. 2020;30(3):485–496.

23. Hotelling H. THE RELATIONS OF THE NEWER MULTIVARIATE STATISTICAL METHODS TO FACTOR ANALYSIS. British Journal of Statistical Psychology. 1957;10(2):69–79. doi:https://doi.org/10.1111/j.2044-8317.1957.tb00179.x.

24. Pearson K. LIII. On lines and planes of closest fit to systems of points in space. The London, Edinburgh, and Dublin Philosophical Magazine and Journal of Science. 1901;2(11):559–572. doi:10.1080/14786440109462720.

25. Jolliffe IT. A note on the use of principal components in regression. Journal of the Royal Statistical Society Series C: Applied Statistics. 1982;31(3):300–303.

26. Liland KH, Mevik BH, Wehrens R. pls: Partial Least Squares and Principal Component Regression; 2023. Available from: `https://CRAN.R-project.org/package=pls`.

27. Höskuldsson A. PLS regression methods. Journal of Chemometrics. 1988;2(3):211–228. doi:https://doi.org/10.1002/cem.1180020306.

28. Abdi H. Partial least square regression (PLS regression). Encyclopedia for research methods for the social sciences. 2003;6(4):792–795.

29. Tay JK, Narasimhan B, Hastie T. Elastic Net Regularization Paths for All Generalized Linear Models. Journal of Statistical Software. 2023;106(1):1–31. doi:10.18637/jss.v106.i01.

30. Tibshirani R. Regression Shrinkage and Selection via the Lasso. Journal of the Royal Statistical Society Series B (Methodological). 1996;58(1):267–288.

31. Habier D, Fernando RL, Kizilkaya K, Garrick DJ. Extension of the bayesian alphabet for genomic selection. BMC Bioinformatics. 2011;12(1):186. doi:10.1186/1471-2105-12-186.

32. Perez P, de los Campos G. Genome-Wide Regression and Prediction with the BGLR Statistical Package. Genetics. 2014;198(2):483–495.

33. Everman ER, McNeil CL, Hackett JL, Bain CL, Macdonald SJ. Dissection of complex, fitness-related traits in multiple Drosophila mapping populations offers insight into the genetic control of stress resistance. Genetics. 2019;211(4):1449–1467.

34. Carbonetto P, Stephens M. Scalable Variational Inference for Bayesian Variable Selection in Regression, and Its Accuracy in Genetic Association Studies. Bayesian Analysis. 2012;7(1):73 – 108. doi:10.1214/12-BA703.

35. Kim Y, Wang W, Carbonetto P, Stephens M. A flexible empirical Bayes approach to multiple linear regression and connections with penalized regression; 2023.

36. Kim Y, Carbonetto P, Stephens M. mr.ash.alpha: Multiple Regression with Adaptive Shrinkage; 2023. Available from: `https://github.com/stephenslab/mr.ash.alpha`.

37. Li Z, Gao N, Martini JW, Simianer H. Integrating gene expression data into genomic prediction. Frontiers in genetics. 2019;10:430679.

38. Orlenko A, Moore JH. A comparison of methods for interpreting random forest models of genetic association in the presence of non-additive interactions. BioData Mining. 2021;14(1):9. doi:10.1186/s13040-021-00243-0.

39. Zeileis A, Hothorn T, Hornik K. Model-Based Recursive Partitioning. Journal of Computational and Graphical Statistics. 2008;17(2):492–514. doi:10.1198/106186008X319331.

40. Marchevsky AM. The Use of Artificial Neural Networks for the Diagnosis and Estimation of Prognosis in Cancer Patients. In: Outcome prediction in cancer. Elsevier; 2007. p. 243–259.

41. Baykal N, Erkmen AM. Resilient backpropagation for RBF networks. In: KES'2000. Fourth International Conference on Knowledge-Based Intelligent Engineering Systems and Allied Technologies. Proceedings (Cat. No. 00TH8516). vol. 2. IEEE; 2000. p. 624–627.

42. Cui T, El Mekkaoui K, Reinvall J, Havulinna AS, Marttinen P, Kaski S. Gene–gene interaction detection with deep learning. Communications Biology. 2022;5(1):1238.

43. Fritsch S, Guenther F, Wright MN. neuralnet: Training of Neural Networks; 2019. Available from: `https://CRAN.R-project.org/package=neuralnet`.

44. Mas JF, Flores JJ. The application of artificial neural networks to the analysis of remotely sensed data. International Journal of Remote Sensing. 2008;29(3):617–663. doi:10.1080/01431160701352154.

45. Edwards SM, Sørensen IF, Sarup P, Mackay TF, Sørensen P. Genomic prediction for quantitative traits is improved by mapping variants to gene ontology categories in Drosophila melanogaster. Genetics. 2016;203(4):1871–1883.

46. Márquez-Luna C, Gazal S, Loh PR, Kim SS, Furlotte N, Auton A, et al. Incorporating functional priors improves polygenic prediction accuracy in UK Biobank and 23andMe data sets. Nature Communications. 2021;12(1):6052.

47. Zheng Z, Liu S, Sidorenko J, Wang Y, Lin T, Yengo L, et al. Leveraging functional genomic annotations and genome coverage to improve polygenic prediction of complex traits within and between ancestries. Nature Genetics. 2024;doi:10.1038/s41588-024-01704-y.

48. Ashburner M, Ball CA, Blake JA, Botstein D, Butler H, Cherry JM, et al. Gene ontology: tool for the unification of biology. Nature genetics. 2000;25(1):25–29.

49. Noah Simon TH Jerome Friedman, Tibshirani R. A Sparse-Group Lasso. Journal of Computational and Graphical Statistics. 2013;22(2):231–245. doi:10.1080/10618600.2012.681250.

50. Simon N, Friedman J, Hastie T, Tibshirani R. A sparse-group lasso. Journal of computational and graphical statistics. 2013;22(2):231–245.

51. Yuan M, Lin Y. Model selection and estimation in regression with grouped variables. Journal of the Royal Statistical Society Series B: Statistical Methodology. 2006;68(1):49–67.

52. McDonald DJ, Liang X, Solón Heinsfeld A, Cohen A. sparsegl: Sparse Group Lasso; 2023. Available from: `https://CRAN.R-project.org/package=sparsegl`.

53. Lee CW, Wilfling F, Ronchi P, Allegretti M, Mosalaganti S, Jentsch S, et al. Selective autophagy degrades nuclear pore complexes. Nature cell biology. 2020;22(2):159–166.

54. Han X, Wang J, Li B, Song Z, Li P, Huang B, et al. Analyses of regulatory network and discovery of potential biomarkers for Korean rockfish (Sebastes schlegelii) in responses to starvation stress through transcriptome and metabolome. Comparative Biochemistry and Physiology Part D: Genomics and Proteomics. 2023;46:101061.

55. Van IJzendoorn SC, Maier O, Van Der Wouden JM, Hoekstra D. The subapical compartment and its role in intracellular trafficking and cell polarity. Journal of cellular physiology. 2000;184(2):151–160.

56. Gergs A, Jager T. Body size-mediated starvation resistance in an insect predator. Journal of Animal Ecology. 2014;83(4):758–768.

57. Privalova V, Labecka AM, Szlachcic E, Sikorska A, Czarnoleski M. Systemic changes in cell size throughout the body of Drosophila melanogaster associated with mutations in molecular cell cycle regulators. Scientific Reports. 2023;13(1):7565.

58. Watson SP, Clements MO, Foster SJ. Characterization of the starvation-survival response of Staphylococcus aureus. Journal of bacteriology. 1998;180(7):1750–1758.

59. Tang H. Regulation and function of the melanization reaction in Drosophila. Fly. 2009;3(1):105–111.

60. Strilbytska OM, Semaniuk UV, Storey KB, Yurkevych IS, Lushchak O. Insulin signaling in intestinal stem and progenitor cells as an important determinant of physiological and metabolic traits in Drosophila. Cells. 2020;9(4):803.

61. Yu Y, Huang R, Ye J, Zhang V, Wu C, Cheng G, et al. Regulation of starvation-induced hyperactivity by insulin and glucagon signaling in adult Drosophila. Elife. 2016;5:e15693.

62. Clark AS, West K, Streicher S, Dennis PA. Constitutive and inducible Akt activity promotes resistance to chemotherapy, trastuzumab, or tamoxifen in breast cancer cells. Molecular cancer therapeutics. 2002;1(9):707–717.

63. Tan X, Lambert PF, Rapraeger AC, Anderson RA. Stress-induced EGFR trafficking: mechanisms, functions, and therapeutic implications. Trends in cell biology. 2016;26(5):352–366.

64. Sevelda F, Mayr L, Kubista B, Lötsch D, van Schoonhoven S, Windhager R, et al. EGFR is not a major driver for osteosarcoma cell growth in vitro but contributes to starvation and chemotherapy resistance. Journal of Experimental & Clinical Cancer Research. 2015;34:1–12.

65. Zacharogianni M, Kondylis V, Tang Y, Farhan H, Xanthakis D, Fuchs F, et al. ERK7 is a negative regulator of protein secretion in response to amino-acid starvation by modulating Sec16 membrane association. The EMBO journal. 2011;30(18):3684–3700.

66. Harvey K, Tapon N. The Salvador–Warts–Hippo pathway—an emerging tumour-suppressor network. Nature Reviews Cancer. 2007;7(3):182–191.

67. Agledal L, Niere M, Ziegler M. The phosphate makes a difference: cellular functions of NADP. Redox Report. 2010;15(1):2–10.

68. Sleiman MSB, Schüpfer F, Lemaitre B, et al. Transforming growth factor β/activin signaling functions as a sugar-sensing feedback loop to regulate digestive enzyme expression. Cell reports. 2014;9(1):336–348.

69. Chng WbA, Koch R, Li X, Kondo S, Nagoshi E, Lemaitre B. Transforming Growth Factor β/Activin signaling in neurons increases susceptibility to starvation. PloS one. 2017;12(10):e0187054.

70. Song W, Cheng D, Hong S, Sappe B, Hu Y, Wei N, et al. Midgut-derived activin regulates glucagon-like action in the fat body and glycemic control. Cell metabolism. 2017;25(2):386–399.

71. Hull-Thompson J, Muffat J, Sanchez D, Walker DW, Benzer S, Ganfornina MD, et al. Control of metabolic homeostasis by stress signaling is mediated by the lipocalin NLaz. PLoS genetics. 2009;5(4):e1000460.

72. Solinas G, Becattini B. JNK at the crossroad of obesity, insulin resistance, and cell stress response. Molecular metabolism. 2017;6(2):174–184.

73. Brown EB, Slocumb ME, Szuperak M, Kerbs A, Gibbs AG, Kayser MS, et al. Starvation resistance is associated with developmentally specified changes in sleep, feeding and metabolic rate. Journal of Experimental Biology. 2019;222(3):jeb191049.

74. Weyrich P, Kapp K, Niederfellner G, Melzer M, Lehmann R, Häring HU, et al. Partitioning-defective protein 6 regulates insulin-dependent glycogen synthesis via atypical protein kinase C. Molecular Endocrinology. 2004;18(5):1287–1300.

75. Consonni SV, Brouwer PM, van Slobbe ES, Bos JL. The PDZ domain of the guanine nucleotide exchange factor PDZGEF directs binding to phosphatidic acid during brush border formation. PLoS One. 2014;9(5):e98253.

76. Humbert PO, Dow LE, Russell SM. The Scribble and Par complexes in polarity and migration: friends or foes? Trends in cell biology. 2006;16(12):622–630.

77. Sherrard KM, Fehon RG. The transmembrane protein Crumbs displays complex dynamics during follicular morphogenesis and is regulated competitively by Moesin and aPKC. Development. 2015;142(10):1869–1878.

78. O'Keefe DD, Prober DA, Moyle PS, Rickoll WL, Edgar BA. Egfr/Ras signaling regulates DE-cadherin/Shotgun localization to control vein morphogenesis in the Drosophila wing. Developmental biology. 2007;311(1):25–39.

79. Paiardi C, Mirzoyan Z, Zola S, Parisi F, Vingiani A, Pasini ME, et al. The stearoyl-CoA desaturase-1 (Desat1) in Drosophila cooperated with Myc to induce autophagy and growth, a potential new link to tumor survival. Genes. 2017;8(5):131.

80. Kučerová L, Kubrak OI, Bengtsson JM, Strnad H, Nylin S, Theopold U, et al. Slowed aging during reproductive dormancy is reflected in genome-wide transcriptome changes in Drosophila melanogaster. BMC genomics. 2016;17:1–25.

81. Harbison ST, Yamamoto AH, Fanara JJ, Norga KK, Mackay TF. Quantitative trait loci affecting starvation resistance in Drosophila melanogaster. Genetics. 2004;166(4):1807–1823.

Click here to access/download;Figure;Fig1.tiff

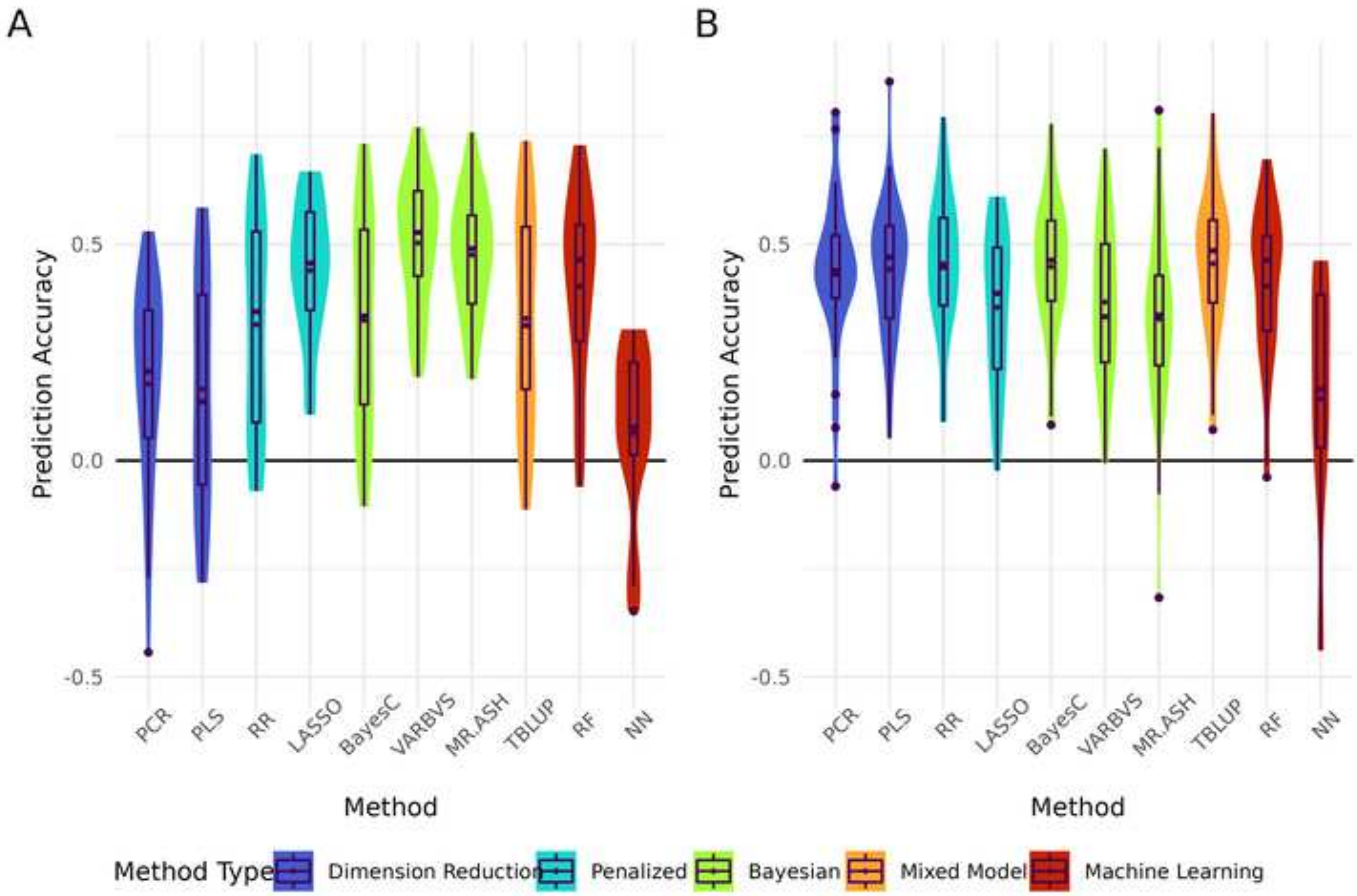

Click here to access/download;Figure;Fig2.tiff

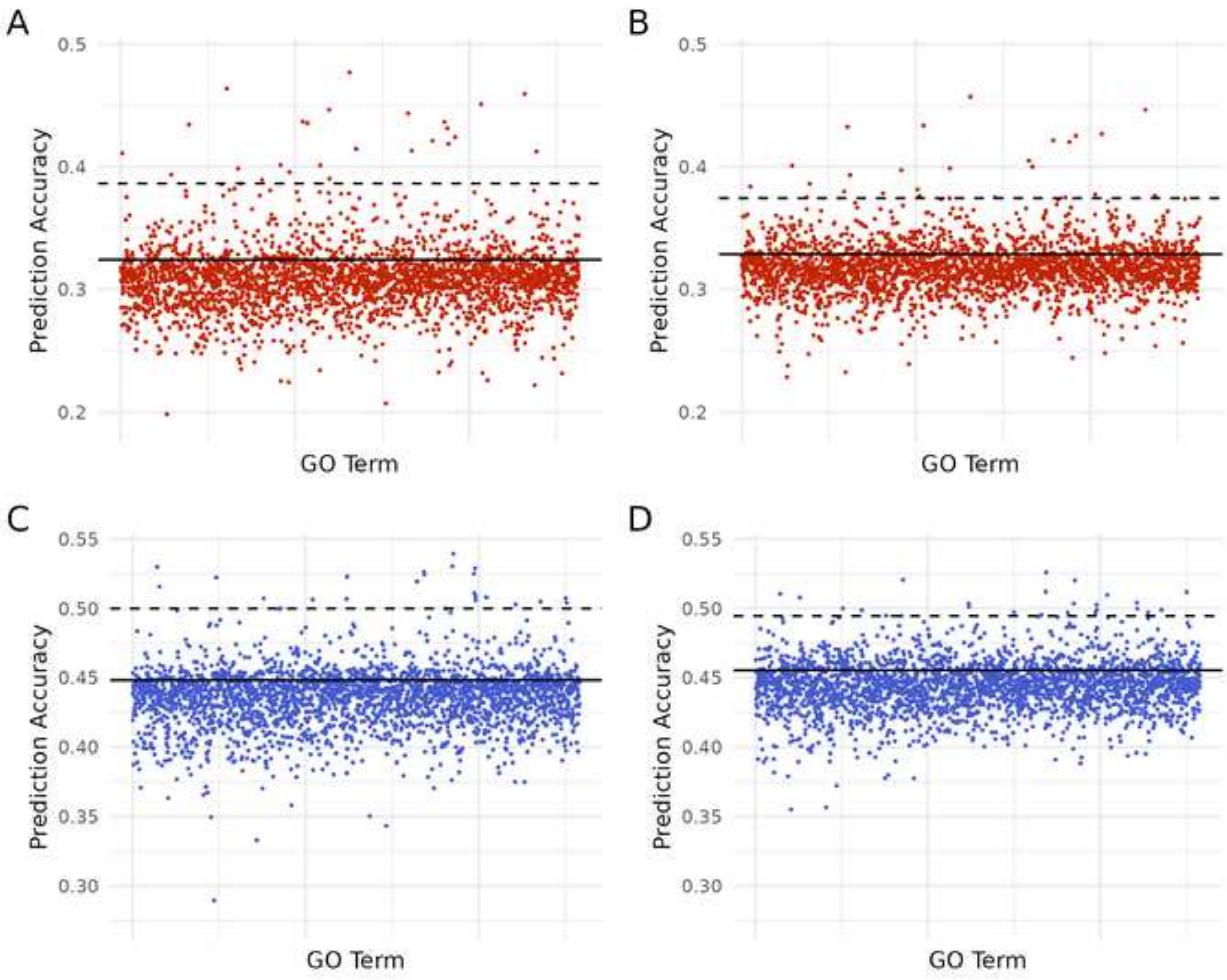

Fig 3

Click here to access/download;Figure;Fig3.tiff

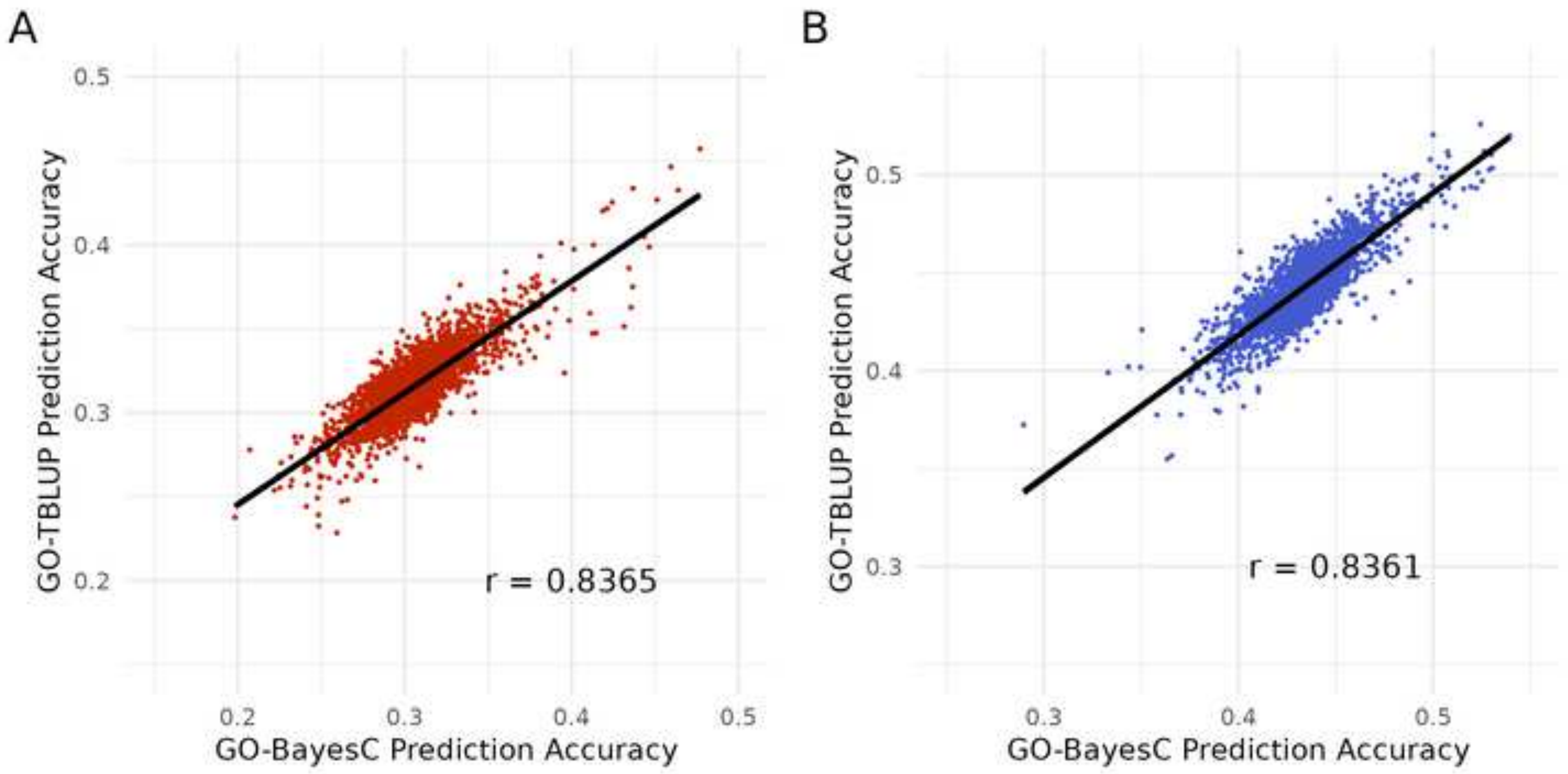

S1 Table

Click here to access/download
Supporting Information
S1_Table.xlsx

S2 Table

Click here to access/download
Supporting Information
S2_Table.xlsx

S3 Table

Click here to access/download
Supporting Information
S3_Table.xlsx

S1 Fig

Click here to access/download
Supporting Information
S1_Fig.tiff

S2 Fig

Click here to access/download
Supporting Information
S2_Fig.tiff
