# Supplementary material for: Comparing statistical learning methods for complex trait prediction from gene expression
Source: PLoS One. 2025 Feb 11;20(2):e0317516. doi: 10.1371/journal.pone.0317516 (PMC11813155; doi:10.1371/journal.pone.0317516)
Supplement: Attachment — Submitted filename: response_to_reviewers.pdf [file pone.0317516.s017.pdf]

Response to Reviewers and Summary of the Revisions

Noah Klimkowski Arango[1,2], Fabio Morgante[1,2*]

1 Center for Human Genetics, Clemson University, Greenwood, SC, USA
2 Department of Genetics and Biochemistry, Clemson University, Clemson, SC, USA

* fabiom@clemson.edu

Summary

We thank the reviewers for their feedback on our manuscript. We made several changes to the manuscript to address the reviewers' comments, which we summarize here:

1. We have added analyses of an additional trait — startle response — with low genetic variation in sexual dimorphism.
2. We have clarified several points made by the reviewers about sample size, prediction accuracy in the $p >> n$ regime, and a few additional points.

We have also highlighted in blue the parts of the main text that have changed substantially in the revised version.

Comments from Reviewer #1

Introduction The study compares statistical learning methods to evaluate the potential of transcriptomic data for accurate prediction of complex traits. For this purpose, the study leverages data from the Drosophila Genetic Reference Panel (DGRP) to evaluate the performance of different prediction models for predicting starvation using transcriptomic data. The study concluded that differences in prediction accuracy are minor and that methods assuming a more polygenic architecture performed better in males whereas methods performing variable selection gave higher accuracy in females. Furthermore, the results show that incorporating functional information (GO terms) can improve prediction accuracy, emphasizing the utility of integrating biological knowledge into statistical models.

Our response We thank the reviewer for taking the time to review our manuscript. This introduction accurately portrays the premise and the key findings of our study.

Comment 1 Strengths: The manuscript provides an extensive comparison of ten statistical methods. By analyzing male and female data separately, the study highlights the importance of considering sex-specific genetic architectures in predictive modeling, which is crucial for traits like starvation resistance that exhibit sexual dimorphism. The results provide a thorough evaluation of differences in prediction accuracy both within sex and between sex. Furthermore, the use of Gene Ontology (GO) annotations to inform prediction models is a significant strength. Lastly, the manuscript acknowledges limitations, such as the small sample size of the DGRP lines, which limits the generalizability of the findings.

Our response We thank the reviewer for the nice words about our study. This comment accurately portrays our key findings about genetic architecture, sex-specificity, the advantage of using functional annotation, and the primary limitation of our work.

Comment 2 The manuscript could comment on similar assessments of statistical methods for prediction of other complex traits based on transcriptomic data and whether these studies yielded similar results.

Our response We thank the reviewer for this suggestion. We followed up our initial results with the analysis of an additional trait, startle response, to address this weakness of our study (see also comment from reviewer #2). For startle response, standard methods that performed variable selection generally had lower prediction accuracy in both sexes. The more similar results between sexes is not unexpected as startle response was chosen because it has low genetic variation for sexual dimorphism. However, differences between sexes in the transcriptome are also present [4], so some level of sex-specificity is still to be expected. GO term analysis and gene analysis confirmed our previous results, whereby a few GO terms are particularly predictive of the trait (chitin binding) and were enriched for biologically relevant genes. This information is included in appropriate sections throughout the manuscript to strengthen our claims.

Comment 3 Furthermore, the manuscript mentions that these methods do not account for non-linear interactions between genes, which could be crucial for predicting complex traits with epistatic effects but does not explore potential solutions to this problem.

Our response We thank the reviewer for this comment. While including epistatic interactions in prediction models has the potential to improve accuracy for some traits, doing so greatly increases the number of effects to estimate. This is very problematic with such a small sample size as the DGRP. For example, prior work using neural networks to detect gene-gene interactions stated that the method is applicable only to large data sets [2]. Having said that, we did use two methods (*i.e.*, random forest and neural networks) that can capture interactions.

The results showed that their performance was not better than the other simpler methods. We have expanded our initial comment in the Discussion to highlight these points.

Comment 4 Additionally, there are some minor issues such as typos (e.g. Lines 240, 259, 291).

Our response We thank the reviewers for catching these typos. We have fixed them.

Comments from reviewer #2

Introduction In this paper by Arango et al, the authors are comparing various statistical models to predict complex phenotypic traits using gene expression data. This question is fundamental to the field as many studies often employ gene expression analysis to identify gene modules using WGCNA/GWAS/TWAS. While the authors approach a very logical question, there is more information needed to strengthen this manuscript.

Our response We thank the reviewer for taking the time to review our manuscript. We just want to clarify that while finding gene modules is certainly an important task, this topic was not investigated in our study.

Comment 1 The authors claim that a limitation of their data is because a sample size of ∼200 cell lines (∼12000 highly expressed genes) is a small sample size, which is not. ∼12000 genes in each sex is a decent sample size to predict phenotypic traits, especially in Drosophila. Thus the authors must speak a bit more on why this is a limitation in the Drosophila field.

Our response We thank the reviewer for this comment. However, we believe that there is some confusion in this comment. First, we did not use *cell* lines in this study. We used *Drosophila melanogaster* inbred lines. These inbred lines were derived from a natural population using 20 generations of full-sib mating. This procedure resulted in minimal genetic variation within each line (*i.e.*, all flies within each line are almost genetically identical) and maximal genetic variation across lines, which reflects the standing genetic variation of the original population from which these lines were derived [1]. Second, when we talk about sample size, we refer to the number of inbred lines ($n = 200$), which are used to estimate the effect sizes of the $p = 12,000$ genes, which are our predictor variables. The effects of the 12,000 genes are then used to predict phenotypes. Estimating 12,000 effects using 200 data points is extremely challenging (the $p >> n$ problem, which is well known in statistics and data science [5]). Thus, we respectfully disagree with the reviewer's comment and reiterate that 200 inbred lines is a small sample size in this context.

Comment 2 The authors themselves mention that the differences in prediction accuracy between the various methods is not large (see abstract and fig 1.). Thus, even if the difference exists, why would it be important to select one method over another if the difference between methods is not large? Moreover, can the authors quantify this difference to accurately determine if the difference is significant or not?

Our response We thank the reviewer for this comment. The sentence in the abstract that the reviewer refers to ("The results show that differences in prediction accuracy between methods exist, although they are generally not large.") is indeed misleading and inaccurate. In fact, the difference in accuracy between some methods is large in a prediction context. Thus, we removed the second part of the sentence, which now reads "The results show that differences in prediction accuracy between methods exist".

In this work, we are interested in broad patterns of differences in prediction accuracy achieved by different methods, not in recommending a method over the others. Because of that and the fact that we have many settings (*i.e.*, 12 methods, 2 sexes, ∼2,500 GO terms), adding results of statistical tests would overcrowd the paper and not be critical for its goals and claims. However, we do provide standard errors of our estimates, for the readers interested in uncertainty measures.

Comment 3 In lines 240-243, the authors mention that the reason the prediction accuracy was low was because of genetic variation in the lines from individual flies. However, even when such methods are applied to human data, we are dealing with high genetic variation among the population. Then in such cases why would any of these methods be employed if their accuracy is low due to genetic heterogeneity? Importantly, for Drosophila in fact the genetic variability will be much lower (considering the lines are inbred; see line 60) than when dealing with human traits and gene expression data. This shows that such methods will ultimately not be able to predict such variable genotypic data with accuracy. The authors should consider commenting on this.

Our response We thank the reviewer for this comment. What we meant with that sentence is that by using the average of many measurements (one for each individual fly) for each line, we essentially increase the broad sense heritability by lowering the environmental variance. This happens because the broad sense heritability of line means is:

$$H_m^2 = \frac{\sigma_G^2}{\sigma_G^2 + \frac{\sigma_E^2}{r}} \tag{1}$$

where σ_G^2 is the genetic variance, σ_E^2 is the environmental variance, and r is the number of flies per line [3].

Thus, a correlation of 0.5, which corresponds to $R^2 = 0.25$, is still pretty far from $H_m^2 = 0.8$, which is typical for *Drosophila* traits assessed on many flies per

line. Again, one of the most likely reasons for this gap is the small sample size of the DGRP.

However, since that sentence did not add significantly to the claims of the paper and had the potential to be confusing, we have decided to remove it.

Comment 4 In this paper the authors select starvation resistance as the phenotypic trait, however the authors state that it is well known that sex differences exists in starvation parameters (see discussion section, line 395-398). When the prediction accuracy of the statistical analysis is low, then why not also select a trait that is not known to differ by sex. This would allow to better understand if sex drives the low accuracy of the statistical parameters or if inherently these statistical tests are poor at predicting complex traits. Thus, the authors should consider comparing the methods with another trait not dependent on sex.

Our response We thank the reviewer for this suggestion. We have added analyses of startle response, a complex behavioral trait for which previous work found low genetic variation in sexual dimorphism (or cross-sex genetic correlation not significantly different from unity) [1]. However, we note that there are still differences between the two sexes in the transcriptome. For example, males and females have different numbers of expressed genes and the expression levels of the majority of those genes showed significant genetic variation in sexual dimorphism [4]. Thus, the possibility of sex-specific results is unavoidable (and is a biologically intrinsic property).

For startle response, standard methods performed similarly in both females and males, suggesting a more similar genetic architecture between sexes. GO term analysis and gene analysis confirmed our previous findings, whereby some terms were particularly predictive of the trait (chitin binding, in the case of startle response). The top genes fell into broader categories of neuron development, sensory receptors, and stress responses.

Minor comments

1. The result section is written more like a discussion section. This is especially true for Gene Analysis. Please consider re-writing this section.
2. Please consider proving a concluding statement for each of your Results sections.

Our response

1. We thank the reviewer for the suggestion. We believe that the information is categorized appropriately, in accordance to the following style. In the Results section, we state functions of GO terms and genes and link them to known systems (*e.g.*, pathways) that are relevant to the trait analyzed, as discovered by previous work (which we cite). In the Discussion section, we compile this information, discuss it in a broader way, and use it to back up the claims made, where appropriate.

2. We thank the reviewer for the suggestion. We have added a concluding statement at the end of each Results subsection.

Comments from reviewer #3

Comment 1 The authors have presented a comparison of various statistical learning methods to predict gene expression. The manuscript is written in a clear and concise manner. The authors though can provide basis to a few parameters used (see attached manuscript). Providing relevance and basis to the parameters used for testing will enhance the depth of their work and provide more context and usefulness to the readers.

Our response We thank the reviewer for this comment. We had originally added the rationale for choosing the R^2 value to the BayesC description only. We have now included this information for all the methods implemented in the BGLR R package (TBLUP, GO-BayesC, GO-TBLUP).

References

[1] Mackay, T., Richards, S., Stone, E., Barbadilla, A., Ayroles, J., Zhu, D., Casillas, S., Han, Y., Magwire, M., Cridland, J. & Others The Drosophila melanogaster genetic reference panel. *Nature.* **482**, 173-178 (2012)

[2] Cui, T., El Mekkaoui, K., Reinvall, J., Havulinna, A., Marttinen, P. & Kaski, S. Gene–gene interaction detection with deep learning. *Communications Biology.* **5**, 1238 (2022)

[3] Falconer, D. Introduction to Quantitative Genetics. (Pearson Education,1996), https://books.google.com/books?id=7ASZNAEACAAJ

[4] Everett, L., Huang, W., Zhou, S., Carbone, M., Lyman, R., Arya, G., Geisz, M., Ma, J., Morgante, F., Armour, G. & Others Gene expression networks in the Drosophila genetic reference panel. *Genome Research.* **30**, 485-496 (2020)

[5] Faraway, J. Linear models with python. (Chapman,2021)
