# Supplementary material for: Comparing statistical learning methods for complex trait prediction from gene expression
Source: PLoS One. 2025 Feb 11;20(2):e0317516. doi: 10.1371/journal.pone.0317516 (PMC11813155; doi:10.1371/journal.pone.0317516)
Supplement: Attachment — Submitted filename: response_to_reviewer.pdf [file pone.0317516.s018.pdf]

Response to Reviewer and Summary of the Revisions

Noah Klimkowski Arango[1,2], Fabio Morgante[1,2*]

1 Center for Human Genetics, Clemson University, Greenwood, SC, USA
2 Department of Genetics and Biochemistry, Clemson University, Clemson, SC, USA

* fabiom@clemson.edu

Summary

We thank the reviewer for their feedback on our manuscript. We made a couple of changes to the manuscript to address the reviewer's comments. We have also highlighted in blue the parts of the main text that have changed substantially in the revised version.

Comments from Reviewer #2

Introduction The authors have decently answered the concerns I raised in the previous version. However, I still have some concerns and believe that it will strengthen the paper.

Our response We thank the reviewer for taking the time to review our manuscript and provide useful suggestions.

Comment 1 While the authors have answered my previous comment regarding editing the result section with their justification, I still believe the part of the results section labeled as "Gene Analysis" has much background information that is not typical to Results sections. Please consider trimming this section down to keep it to the results and move parts to discussion session especially where previous studies have been compared.

Our response We thank the reviewer for this suggestion. We have trimmed the Gene Analysis section in the main text, moving the detailed description of the genes and their relevance to the traits to the S1 Text. Since this is mainly a prediction paper, we did not want to add too many non-prediction details to the Discussion section, risking to dilute the key points.

Comment 2 I feel like that the authors removed the lines in 240-243 in the previous version on the basis of it not being significant to their claim and the potential for it to be confusing (mentioned in the response to the reviewer letter), but I in fact think that it is valuable when papers provide limitations of their

study as no study is perfect. My suggestion would be to add this statement back and explain with the statistical equation (provided in the response letter) that the authors put in reviewer comments which will back their reasoning and avoid any confusion.

Our response We thank the reviewer for this suggestion. We have added the statement back to the manuscript and included the explanation.
